# A Trajectory-Regularized Physics-Informed Hybrid Framework for Specialty Fresh Food Commodity Price Forecasting and Market Stability Monitoring

**DOI:** 10.3390/foods15132305

**Published:** 2026-06-29

**Authors:** Fengyu Li, Yujie Li, Xingyu Gao, Qimiao Wang, Wenzhe Yuan, Qinyou Sun, Yanan Gao, Shaoteng Gao, Ke Zhu, Jun Yan, Pingzeng Liu, Xianyong Meng

**Affiliations:** 1 College of Information Science and Engineering, Shandong Agricultural University, Tai’an 271018, China; 2023121117@sdau.edu.cn (F.L.); 2023121133@sdau.edu.cn (Y.L.); 2024121194@sdau.edu.cn (X.G.); 2024121209@sdau.edu.cn (Q.W.); 2024121218@sdau.edu.cn (W.Y.); 2024121207@sdau.edu.cn (Q.S.); 2023121123@sdau.edu.cn (Y.G.); 2024121193@sdau.edu.cn (S.G.); kezhu@sdau.edu.cn (K.Z.); yanj2016@sdau.edu.cn (J.Y.); 2 Key Laboratory of Huanghuaihai Smart Agricultural Technology, Ministry of Agriculture and Rural Affairs, Tai’an 271018, China; 3 Agricultural Big Data Research Center, Shandong Agricultural University, Tai’an 271018, China

**Keywords:** fresh food commodities, specialty crop prices, food commodity price forecasting, food market stability, trajectory regularization, physics-informed learning, multi-source data fusion

## Abstract

Price volatility in fresh food commodities can weaken supply-chain coordination, disturb market expectations, and increase short-term risks to food availability and affordability. This issue is more pronounced for specialty crops with seasonal production, concentrated supply, limited storability, and high sensitivity to climate, trade, energy, and online-attention shocks. This study develops a trajectory-regularized physics-informed multi-source forecasting framework for daily wholesale prices of garlic, scallion, and ginger in China from 2014 to 2024. The framework, denoted as STL–ETO–EMA–PILSTM, integrates Seasonal-Trend decomposition using LOESS (STL), Efficient Multi-scale Attention (EMA), Long Short-Term Memory (LSTM), an economically motivated physics-informed trajectory residual constraint, and Exponential-Trigonometric Optimization (ETO), using production, climate, macroeconomic, trade, crude-oil, and online-attention indicators. In this framework, the physics-informed component is implemented as a trajectory residual constraint inspired by price-adjustment inertia and local continuity, rather than as a conventional PINN based on strict governing physical equations. In one-step-ahead forecasting, the model outperformed conventional machine learning baselines and additional time-series baselines, including naive persistence, Transformer Encoder, and PatchTST, with MAE values of 0.0853, 0.0581, and 0.1409 for garlic, scallion, and ginger, respectively, and R^2^ values above 0.996. Leakage-prevention procedures, walk-forward validation, multi-horizon forecasting, and Diebold–Mariano tests were used to strengthen result credibility. Multi-step forecasting showed clear performance degradation as the horizon increased, supporting the positioning of the framework as a short-term market-monitoring tool rather than a long-horizon structural projection model. Permutation-based feature-importance and interaction analyses revealed crop-specific price drivers. The framework provides an interpretable tool for fresh food price forecasting, market stability monitoring, and short-term operational risk monitoring in fresh food supply chains.

## 1. Introduction

Accurate forecasting of food commodity prices supports market regulation, public information provision, procurement planning, and short-term risk awareness in agri-food systems. In recent years, adaptive food-price forecasting and uncertainty-aware prediction have become increasingly important when food markets face rapid economic change, pandemic-related disruptions, geopolitical shocks, and inflationary pressure [1]. Deep-learning models driven by exogenous variables have also been shown to improve agricultural crop price forecasting by incorporating weather, market, and macroeconomic information [2]. Garlic, scallion, and ginger are widely consumed fresh seasoning commodities in China, and their prices are affected by seasonal supply cycles, geographically concentrated production, limited storability, circulation conditions, and short-term market expectations [3]. These characteristics make them suitable representative commodities for examining short-term specialty fresh food price forecasting and market stability monitoring.

Price movements in fresh food and agricultural commodities are shaped by interacting supply, demand, climate, macroeconomic, trade, and information-related factors [2,4]. Recent studies show that incorporating exogenous variables, such as weather, market indicators, and macroeconomic signals, can improve agricultural price forecasting, especially for perishable or highly volatile crops [2]. At the same time, major disruptions, including COVID-19, geopolitical conflicts, and the Russia–Ukraine war, have increased food-price uncertainty through trade disruption, cost transmission, energy-market pressure, and changing market expectations [1,4,5]. When these forces interact, the resulting price process is often nonlinear, non-stationary, and difficult to forecast with conventional tools [6]. China-specific trade and policy variables were retained as contextual indicators because the empirical application focuses on Chinese specialty crop markets [7].

Earlier studies on agricultural and food commodity price forecasting mainly used econometric models, feature-based machine learning methods, and forecast combinations. These approaches provide useful baselines, but they may be less effective when price series become nonlinear, non-stationary, and strongly affected by abrupt market shocks [6]. Recent studies have therefore introduced optimized machine learning and hybrid deep-learning frameworks for agricultural commodity price prediction [6,8]. These methods improve modeling flexibility, but capturing sequential dependence, hidden market states, and shifting temporal interactions remains challenging in highly volatile price series [4,6].

Recent international forecasting studies have also moved beyond conventional recurrent neural networks. Transformer-based models and attention-enhanced architectures have been introduced for multi-commodity agricultural price forecasting and anomaly detection, showing stronger capacity to capture long-range dependence and sudden market changes [9]. Exogenous-variable-driven deep-learning models, including TransformerX and NBEATSX, further show that weather and market-related inputs can improve price forecasting for perishable crops [2]. In parallel, probabilistic and adaptive food-price forecasting has become increasingly important because point forecasts alone may not provide sufficient information for decision-making under rapid economic change [1]. Explainable forecasting frameworks have also received growing attention, as food-market monitoring requires not only accurate prediction but also transparent interpretation of market drivers [10]. However, higher numerical accuracy alone does not solve all difficulties in specialty-crop forecasting. Garlic, scallion, and ginger prices are often affected by abrupt shocks, seasonal changes, and structural shifts. Models trained only by fitting data may become sensitive to noise and unstable across market phases, and may still generate trajectories that are difficult to interpret from the perspective of market adjustment and short-term monitoring. Although recent studies have advanced agricultural price forecasting through optimized machine learning, Transformer-based models, attention mechanisms, and explainable forecasting [8,9,10], most existing work still focuses on either numerical accuracy, anomaly detection, or model-specific interpretability. Fewer studies have jointly considered structural decomposition, multi-scale temporal representation, economically informed trajectory regularization, uncertainty-aware monitoring, and cross-crop market-driver interpretation within a unified short-term fresh food market monitoring framework [1,9,10].

The multi-scale structure of specialty-crop prices further complicates forecasting because short-term volatility, medium-term cyclical adjustment, and long-term structural movement are often mixed in the same series. A single sequential model may struggle to capture these temporal patterns in a stable way. This motivates the use of hybrid frameworks that combine structural decomposition, temporal representation learning, and trajectory regularization.

This has led to more hybrid forecasting frameworks that combine decomposition, feature selection, optimization, and deep learning. Recent studies have advanced agricultural price forecasting through optimized machine learning, Transformer-based models, attention mechanisms, and explainable forecasting [8,9,10]. Even so, many hybrid models still mainly follow a data-fitting logic. Domain knowledge is rarely embedded in the training objective, and the resulting forecasts may remain difficult to interpret from the perspectives of market adjustment, price formation, and short-term food-market monitoring.

Physics-informed learning provides a useful idea for incorporating structural or residual constraints into data-driven forecasting models. In conventional PINNs, such constraints are often derived from explicit governing equations. In this study, however, food commodity prices are not assumed to follow a strict physical law or a complete mechanistic market equation. The term “physics-informed” is therefore used in a broader constraint-guided sense. Specifically, the PILSTM component introduces a second-order trajectory residual into the LSTM training objective. This residual approximates the local curvature of the forecast path and is motivated by price-adjustment inertia, market-adjustment continuity, transaction frictions, delayed information diffusion, and the avoidance of economically unsupported high-frequency reversals in short-term wholesale price movements. This design follows the general physics-informed learning principle of combining a data-fitting loss with a residual-based structural constraint, but the residual term is economically motivated and trajectory-based rather than derived from a physical governing equation. In this way, the framework uses domain-informed trajectory regularization to improve forecast-path plausibility while retaining the flexibility of data-driven sequence learning.

For agri-food market analytics and food-system resilience, the central challenge is not only to reduce forecasting error, but also to provide stable short-term price trajectories, integrate multi-source market signals, and identify commodity-specific drivers behind price fluctuations. Specialty crop prices are not single-scale signals; they combine long-term structural movement, medium-term cyclical adjustment, and short-term local disturbance within the same temporal process. Although recent studies have advanced agricultural price forecasting through optimized machine learning, Transformer-based models, attention mechanisms, and explainable forecasting [8,9,10], most existing work still focuses on either numerical accuracy, anomaly detection, or model-specific interpretability. Fewer studies have jointly considered structural decomposition, multi-scale temporal representation, economically informed trajectory regularization, uncertainty-aware monitoring, and cross-crop market-driver interpretation within a unified short-term fresh food market monitoring framework [1,9,10]. This gap is particularly important for specialty fresh food commodities, whose prices may respond rapidly to production changes, trade conditions, energy costs, information-related market signals, and circulation pressure.

The methodological contribution of this study should be understood from the perspective of task-oriented integration rather than from the invention of isolated algorithms. STL, EMA, LSTM, ETO, and regularization-related ideas have been used in previous forecasting studies. However, existing hybrid decomposition–deep learning models often emphasize numerical accuracy, while paying less attention to the joint requirements of non-stationary price decomposition, short-term monitoring, forecast-path stability, and crop-specific market-driver interpretation in specialty fresh food markets. To address this gap, this study organizes these existing and adapted components into a coordinated forecasting framework. In the proposed framework, STL reduces the complexity of non-stationary price movements by separating structural components; EMA refines multi-scale temporal representations within the sliding window; the physics-informed trajectory-regularized constraint improves forecast-path plausibility by penalizing economically unsupported local oscillations; ETO provides lightweight offline model-configuration selection; and permutation-based interpretation identifies commodity-specific predictive drivers. Therefore, the novelty of the framework lies in its coordinated design for short-term specialty fresh food commodity price monitoring, rather than in any single module alone.

Against this background, this study develops a hybrid framework integrating Seasonal-Trend decomposition using LOESS (STL), Exponential-Trigonometric Optimization (ETO), Efficient Multi-scale Attention (EMA), and a physics-informed-style LSTM with an economically motivated trajectory residual constraint. The proposed framework is abbreviated as STL–ETO–EMA–PILSTM. STL separates raw price series into trend, seasonal, and residual components; EMA refines multi-scale temporal representations; PILSTM improves forecast-path stability through trajectory residual regularization; and ETO is used as an offline model-configuration search strategy. Using garlic, scallion, and ginger as representative specialty fresh food commodities, this study evaluates whether the framework can improve forecasting accuracy, trajectory stability, robustness, and interpretability under heterogeneous fluctuation regimes.

The main contributions of this study are as follows:

(1) Hybrid computational architecture for non-stationary food commodity time series.

A task-oriented hybrid framework is developed by coordinating structural decomposition, multi-scale temporal feature refinement, economically motivated physics-informed trajectory regularization, sequence learning, and offline model-configuration selection into a coherent forecasting workflow. Rather than simply stacking existing methods, the framework assigns each component a specific role in modeling volatile specialty fresh food commodity prices: structural decomposition reduces the complexity of non-stationary price movements, multi-scale temporal refinement strengthens short-term and medium-term representation, the trajectory-regularized constraint improves forecast-path stability, and offline optimization supports robust model configuration. This architecture is designed to improve short-term forecasting robustness and market-monitoring relevance for specialty fresh food commodity price series.

(2) Decomposition-enhanced multi-scale temporal representation.

A decomposition–attention coupling strategy is introduced by integrating the Efficient Multi-scale Attention (EMA) module to model long-term movement, cyclical fluctuation, and short-term disturbance in specialty fresh food commodity prices. This design improves the retention and aggregation of temporal information across different scales.

(3) Physics-informed trajectory-regularized PILSTM module for forecast-path plausibility

A PILSTM module is developed by embedding a second-order trajectory residual into the LSTM training objective. The module retains the residual-constrained training logic of physics-informed learning, but it does not assume that food commodity prices follow strict physical governing equations. Instead, the residual term is motivated by price-adjustment inertia, market-adjustment continuity, and local smoothness in short-term wholesale markets, and is used to reduce economically unsupported local oscillations in the predicted path.

(4) Lightweight offline model-configuration selection for coupled hybrid models.

An Exponential-Trigonometric Optimization (ETO)-based search strategy is used to select an empirically suitable model configuration for the coupled hybrid framework under a fixed computational budget. This procedure reduces dependence on manual tuning and improves the reproducibility and stability of the model configuration, but it should not be interpreted as an exhaustive global hyperparameter optimization process.

(5) Cross-crop validation on heterogeneous specialty food commodity price series.

Experiments on garlic, scallion, and ginger show that the proposed framework performs consistently across crops with different fluctuation patterns, supporting its use in non-stationary specialty crop forecasting and short-term food market monitoring.

## 2. Materials and Methods

This section describes the study design, data sources, variable construction, preprocessing procedure, forecasting framework, baseline models, and evaluation metrics. The proposed framework was designed for short-term price forecasting of specialty fresh food commodities under non-stationary and volatile market conditions. The workflow included multi-source data collection, preliminary variable analysis, sample construction, STL–ETO–EMA–PILSTM model development, hyperparameter setting, and model evaluation.

### 2.1. Study Design

This study used garlic, scallion, and ginger as representative specialty fresh food commodities. These commodities were selected not only because of their price volatility, but also because they are widely consumed seasoning-type fresh food commodities in China and play a direct role in household consumption and catering supply. They share several market characteristics, including seasonal production, geographically concentrated producing areas, limited storability, and sensitivity to climate, circulation, trade, macroeconomic conditions, and information-related shocks. At the same time, they differ in market-transmission mechanisms: garlic is more closely linked to export demand, scallion is more sensitive to domestic supply and circulation conditions, and ginger shows a more mixed response to supply, trade, macroeconomic, and information-related factors. This combination provides a heterogeneous empirical setting for examining whether the proposed framework can remain effective across different volatility patterns and market-transmission mechanisms within Chinese specialty fresh food markets.

The forecasting task was defined as one-step-ahead daily wholesale price prediction. Given a fixed historical window, the model used past prices and multi-source explanatory variables available before the forecasting date to predict the next-day price. This setting is intended for short-term food-market surveillance and operational risk monitoring. Its practical use lies in daily price tracking, procurement timing, replenishment planning, inventory adjustment, and early detection of abnormal price movements. It is not intended to replace medium- or long-term production planning, acreage adjustment, policy projection, or strategic supply-chain optimization.

### 2.2. Data Sources, Price Series, and Forecasting Task

Daily wholesale prices of garlic, scallion, and ginger from 1 January 2014 to 31 December 2024 were obtained from the National Key Agricultural Products Market Information Platform of the Ministry of Agriculture and Rural Affairs of China. After calendar alignment, the final dataset contained 4018 daily observations for each commodity. The target variables were the daily wholesale prices of garlic, scallion, and ginger. The use of an official institutional source helps ensure data consistency and reliability.

To construct a multi-source forecasting framework, explanatory variables were introduced to characterize supply conditions, macroeconomic environments, international market transmission, energy-related costs, and information-related influences. These variables include temperature in major producing regions, planting area, production volume, fresh vegetable consumer price index, broad money supply, exchange rate, international crude-oil prices, export volume, export value, and online public attention indices. These explanatory variables were collected at daily, monthly, or annual frequencies and were temporally aligned to the daily forecasting calendar according to their actual availability before the forecasting date.

Export value and export volume were incorporated as lagged variables to reflect delayed market transmission from external demand conditions. Daily variables were lagged when their same-day values would not be available before prediction. Monthly and annual variables were carried forward only after their official availability. Only historical values available before the forecasting date were used as model inputs, and no contemporaneous target information was introduced into the forecasting process. This setting helps avoid target leakage and preserves the temporal consistency of the prediction task.

**Figure 1:**
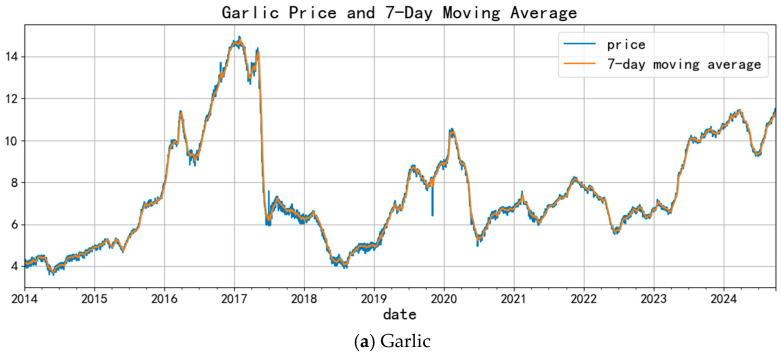

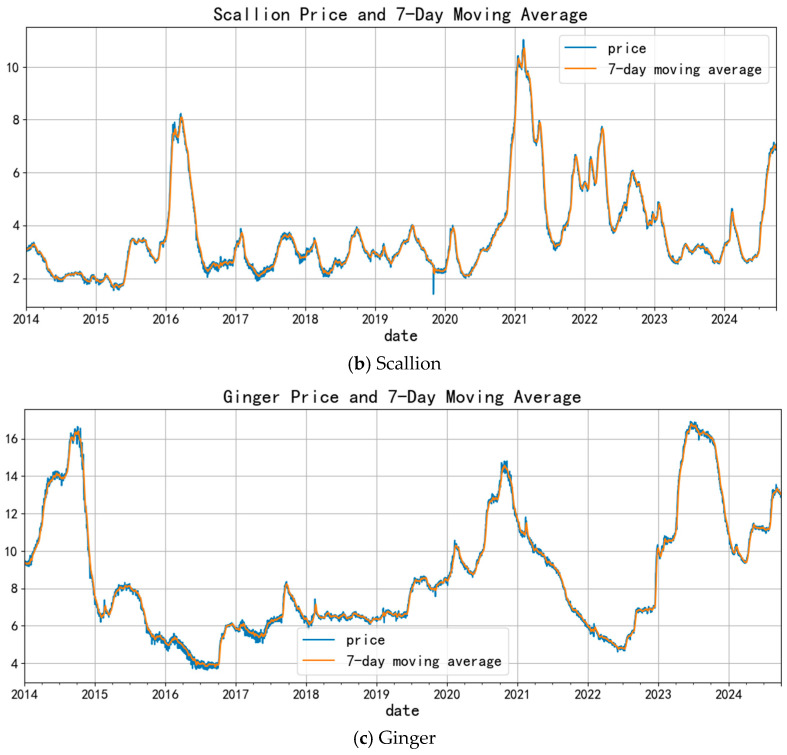
Price series and 7-day moving averages for garlic, scallion, and ginger.

A 7-day moving average was used only for descriptive visualization of the original price series. It was not used to replace the raw price series in model training. As shown in Figure 1, the three commodities exhibit recurring fluctuations, longer-term movement, and abrupt local changes. These characteristics support the use of decomposition-based and multi-scale forecasting methods.

A sliding-window strategy was used to transform the original time series into supervised learning samples. In the main experiment, a 30-day input window was used, and each sample used the previous 30 days of observations to predict the next-day wholesale price, corresponding to a one-step-ahead forecasting task. The dataset was divided chronologically into 80% training data and 20% testing data to preserve the temporal order of the series and avoid information leakage caused by random splitting.

### 2.3. Variable Construction and Preliminary Relationship Analysis

The variable system was constructed according to economic relevance, empirical support, data availability, and temporal consistency. Historical prices were used to capture price inertia and short-term persistence. Supply-side variables, including planting area, production volume, temperature, and agricultural production cost indicators, were used to describe production capacity and environmental conditions. Macroeconomic variables, including the fresh vegetable consumer price index, broad money supply, and exchange rate, were used to characterize broader demand, liquidity, and cost-transmission effects. International market variables, including export value, export volume, and crude-oil prices, were introduced to capture trade demand and energy-related cost pressure. Policy, shock, and online-attention variables were included to reflect external disturbances and information-driven market expectations. Trade- and shock-related variables were used to characterize external demand, geopolitical disruption, and cost-transmission pressure [1,4,5,7], while online-attention variables were used to capture information diffusion and short-term market expectations in vegetable and specialty agricultural product markets [11,12].

Related agricultural and food price forecasting studies have used forecast combinations, optimization-based models, ARIMA–LSTM hybrids, crop-specific trend prediction, memory-based neural networks, and artificial neural networks in comparable commodity-price settings [13,14,15,16,17,18]. Recent hybrid and decomposition-based studies have also combined VMD–LSTM structures, federated transfer learning, STL–attention–LSTM, recursive feature selection, and deep learning to improve forecasting performance for nonlinear and non-stationary agricultural or commodity price series [19,20,21,22,23,24,25]. The multi-source variables used in the forecasting framework are summarized in Table 1.

**Figure 2:**
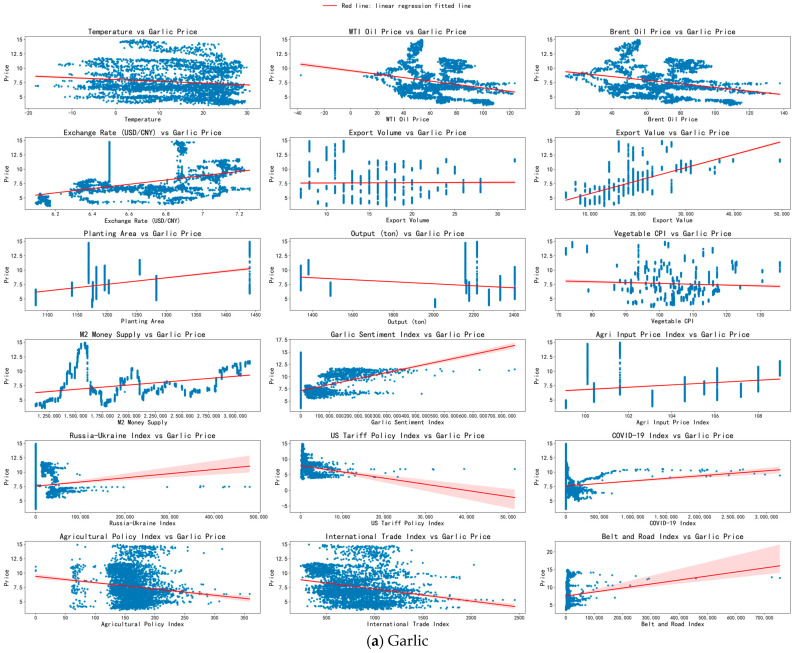

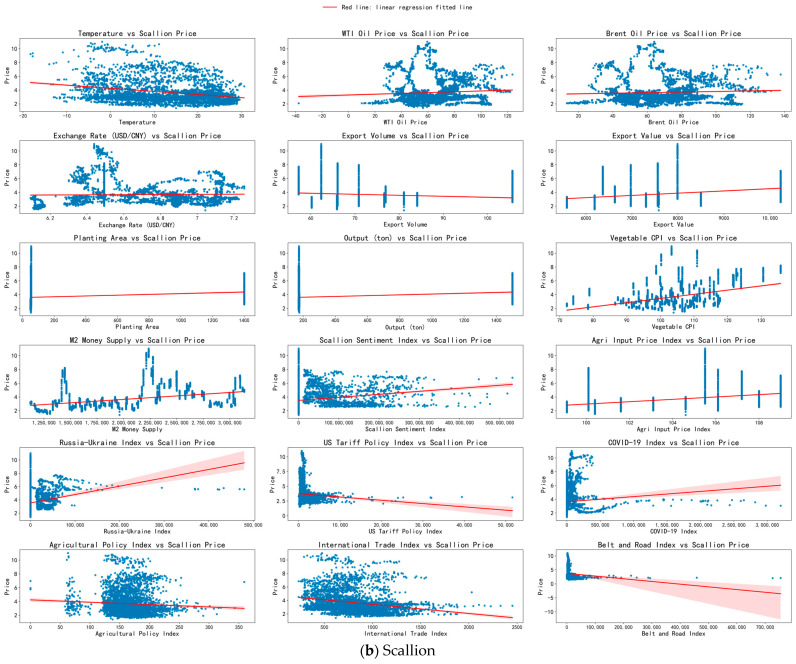

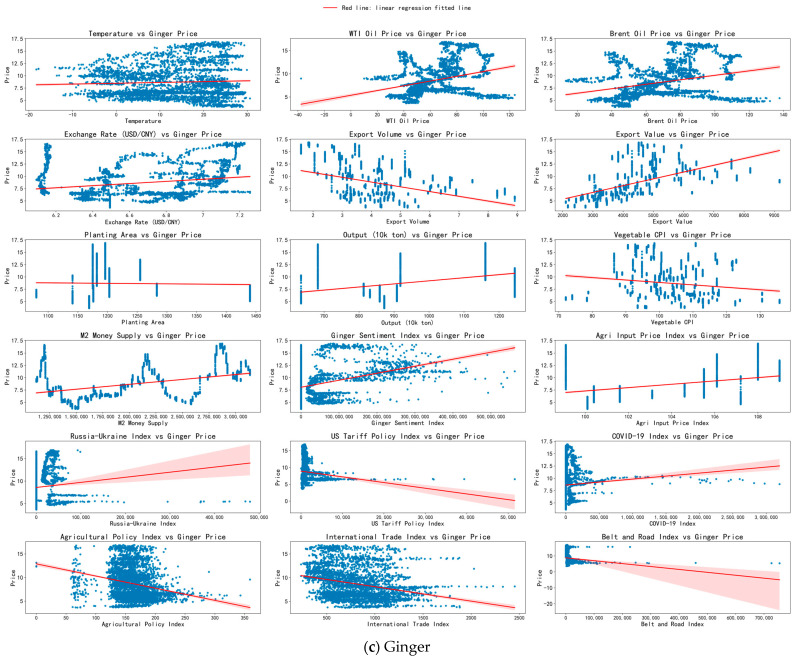
Regression smoothing plots between commodity prices and selected influencing factors for garlic, scallion, and ginger. The red line denotes the fitted smoothing trend.

Regression smoothing plots were used to examine possible nonlinear associations between commodity prices and explanatory variables. These plots were used for exploratory relationship analysis and variable screening, not for causal identification. As shown in Figure 2, the fitted relationships are crop-specific and often nonlinear. Garlic prices show clearer associations with export-related and expectation-related variables, scallion prices respond more strongly to domestic supply and macroeconomic variables, and ginger prices display a broader and less stable set of associations.

**Figure 3:**
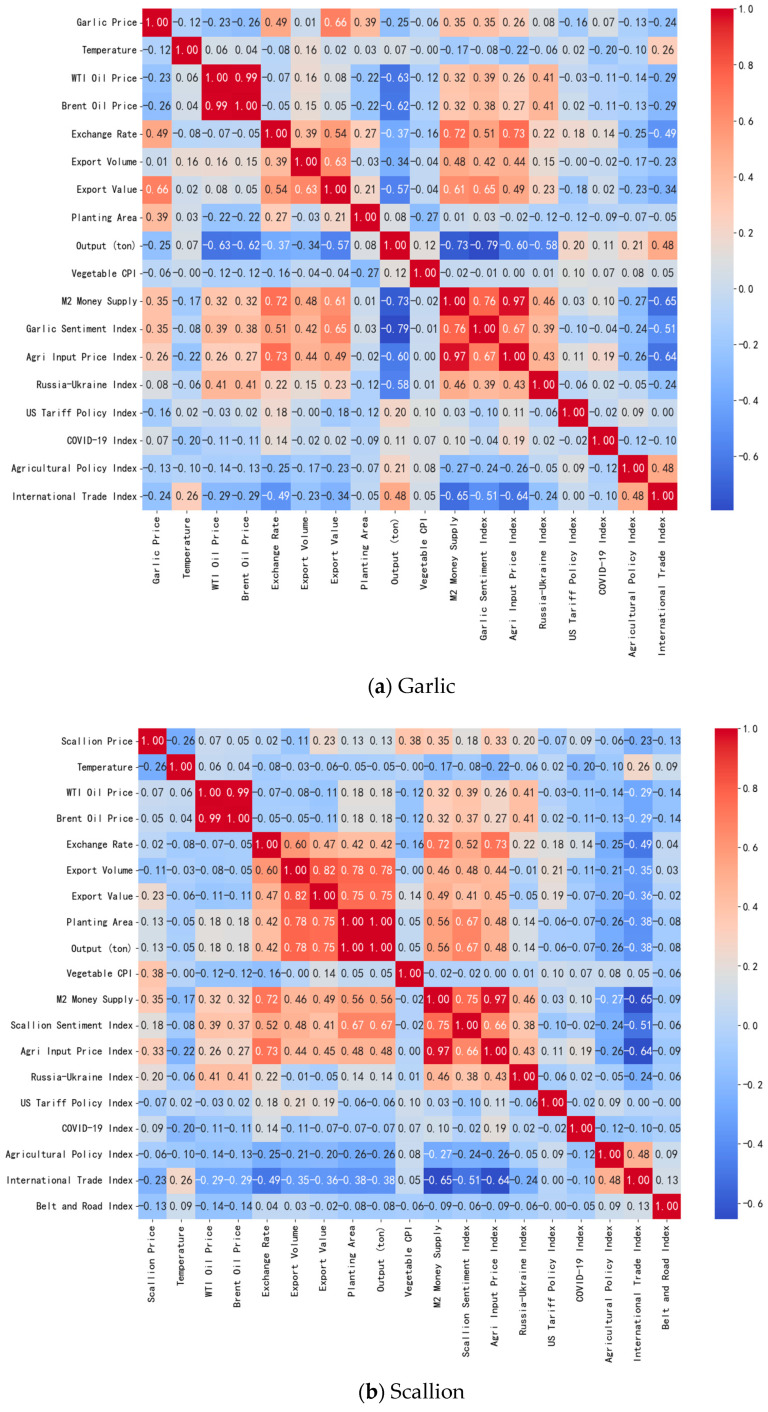

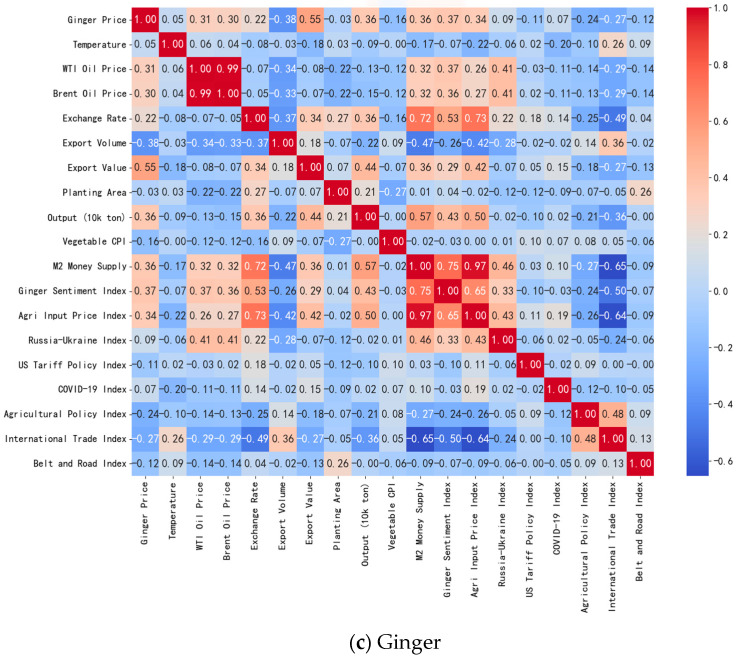
Correlation heatmaps between influencing factors and prices of garlic, scallion, and ginger.

Pearson correlation heatmaps were then used to examine the internal dependence structure among explanatory variables and their linear associations with price. As shown in Figure 3, the explanatory system contains grouped dependence, especially among macroeconomic and external-market indicators. The three commodities also show different correlation structures, suggesting that a uniform linear model may be insufficient for all forecasting tasks.

The preliminary analysis indicates that the forecasting framework should be able to handle mixed temporal components, nonlinear variable–price relationships, and commodity-specific driver structures. This provides the basis for using structural decomposition, multi-scale temporal representation, and trajectory regularization in the proposed model.

### 2.4. Data Preprocessing and Sample Construction

Data preprocessing included duplicate removal, outlier checking, missing-value treatment, temporal alignment, normalization, STL decomposition, and supervised sample construction. Duplicated records and visibly abnormal values were checked before model training. Missing values were treated according to variable frequency and economic meaning. For daily continuous variables, short missing intervals were filled by linear interpolation. For monthly and annual variables, the most recently available value was carried forward until a new officially released value became available. Obvious recording errors were examined against adjacent observations and source consistency. Real market shocks, such as COVID-19, trade frictions, geopolitical events, logistics disruptions, and other abnormal market disturbances, were not removed because they represent genuine market conditions that a food-market monitoring model should learn to reflect.

All numerical variables were normalized to improve training stability and convergence. To avoid information leakage, normalization parameters were estimated from the training subset only and then applied to the validation and testing subsets. After prediction, output values were transformed back to the original price scale for evaluation.

The supervised learning samples were constructed using a sliding-window strategy. Let L denote the input window length. For each time point t, the input contained the historical observations from t−L+1 to t and the target was the next-day price $y_{t+1}$. The supervised sample can be represented as:

(1) $$X_{t}=[z_{t-L+1},z_{t-L+2},\ldots ,z_{t}]$$

(2) $${\hat{y}}_{t+1}=F(X_{t};\theta )$$

where $z_{t}$ denotes the structured input features at time t, ${\hat{y}}_{t+1}$ is the predicted price, F(•) is the forecasting model, and θ denotes model parameters.

For each target value, the input window contained only historical prices and explanatory variables observed or officially available before the forecasting date. Annual production-related indicators were represented by the most recently available annual statistics rather than by future annual totals. Monthly indicators, such as CPI, M2, export value, and export volume, were aligned according to their reporting frequency and carried forward only after official availability. Online-attention indicators were lagged by one day to reduce the risk of using same-day information that would not be available before prediction.

### 2.5. Proposed STL–ETO–EMA–PILSTM Framework

To handle the non-stationary and multi-scale characteristics of specialty fresh food commodity prices, this study developed a hybrid forecasting framework termed STL–ETO–EMA–PINN–LSTM. Hereafter, the framework is abbreviated as STL–ETO–EMA–PILSTM. The framework combines STL-based structural decomposition, EMA-based multi-scale temporal representation, an economically motivated physics-informed trajectory-regularized LSTM module for forecast-path control, and ETO-based offline model-configuration selection.

**Figure 4:**
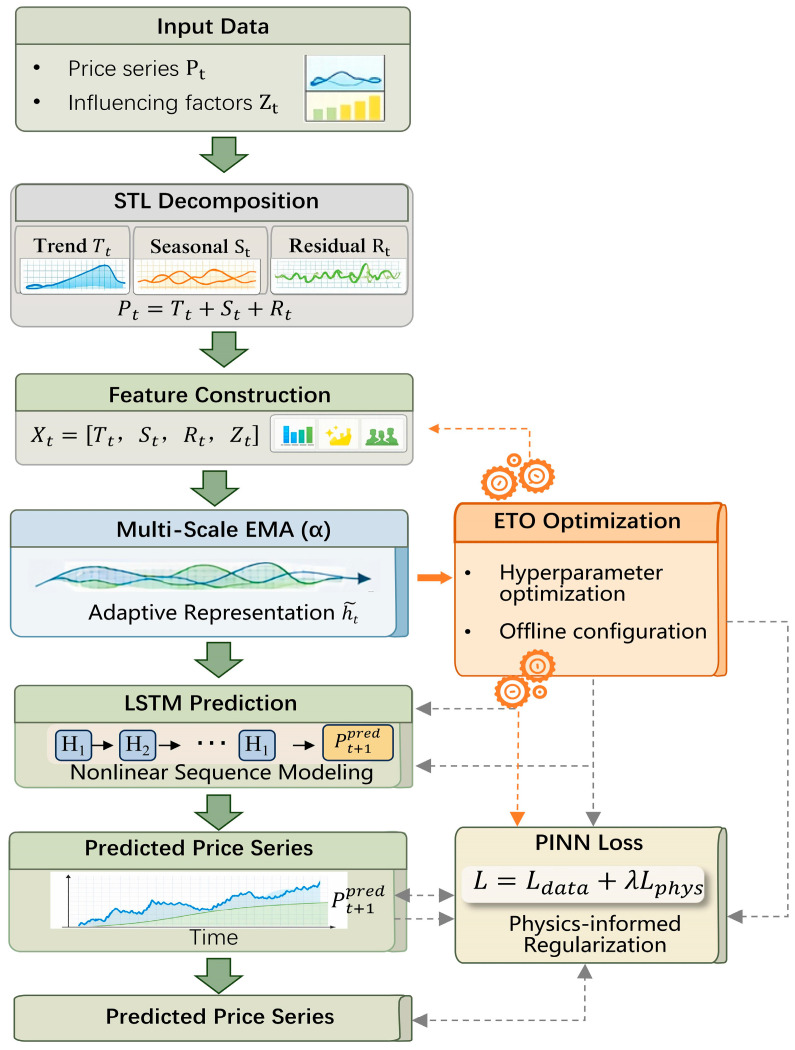
Architecture of the proposed STL–ETO–EMA–PILSTM framework. Seasonal-Trend decomposition using LOESS (STL) extracts structural components from raw price series, Efficient Multi-scale Attention (EMA) generates multi-scale temporal representations, Long Short-Term Memory (LSTM) performs nonlinear sequence modeling, the physics-informed trajectory-regularized LSTM component imposes an economically motivated trajectory residual constraint through the training loss, and Exponential-Trigonometric Optimization (ETO) is used for offline model-configuration selection.

As shown in Figure 4, the framework first decomposes the raw price series into trend, seasonal, and residual components. The decomposed components are then combined with multi-source explanatory variables to form structured input features. EMA refines temporal representations across different time scales. The PILSTM module performs sequence prediction under an economically motivated physics-informed trajectory-regularized constraint. ETO is used to search for suitable hyperparameters before final model training.

#### 2.5.1. Framework Architecture

Let $y_{t}$ denote the observed price at time t, and let $x_{t}$ denote the vector of explanatory variables. The overall framework can be written as:

(3) $$z_{t}=\Phi (\mathrm{STL}(y_{t}),x_{t})$$

(4) $$h_{t}=\mathrm{EMA}(z_{t})$$

(5) $${\hat{y}}_{t+1}=\mathrm{PILSTM}(h_{t-L+1:t})$$

where STL(•) decomposes the price series, Φ(•) constructs structured input features, EMA(•) refines temporal representations, and PILSTM(•) denotes the physics-informed trajectory-regularized LSTM predictor.

ETO is not part of the forward prediction process. It is used only as an offline model-configuration search strategy before final training. After the final configuration is fixed, prediction is performed by the STL, EMA, and physics-informed trajectory-regularized LSTM components.

#### 2.5.2. STL-Based Structural Decomposition

Specialty fresh food commodity prices often contain long-term movement, recurring seasonal fluctuation, and irregular local disturbance in the same sequence. If these components remain mixed, the predictor may struggle to distinguish persistent changes from temporary shocks. Seasonal-Trend decomposition using LOESS (STL) was therefore introduced to separate the raw price series into more interpretable components [26].

Before applying STL decomposition, the price series were inspected using seasonal patterns, autocorrelation behavior, and decomposition diagnostics. The three commodities showed recurring seasonal movements together with irregular local disturbances, which supports the use of STL to separate trend, seasonal, and residual components. STL was used as a structural preprocessing tool rather than as evidence that the series are strictly stationary.

The observed price series is decomposed as:

(6) $$y_{t}=T_{t}+S_{t}+R_{t}$$

where $T_{t}$, $S_{t}$, and $R_{t}$ denote the trend, seasonal, and residual components, respectively.

After decomposition, the structured input vector is defined as:

(7) $$z_{t}=[T_{t},S_{t},R_{t},x_{t}]$$

**Figure 5:**
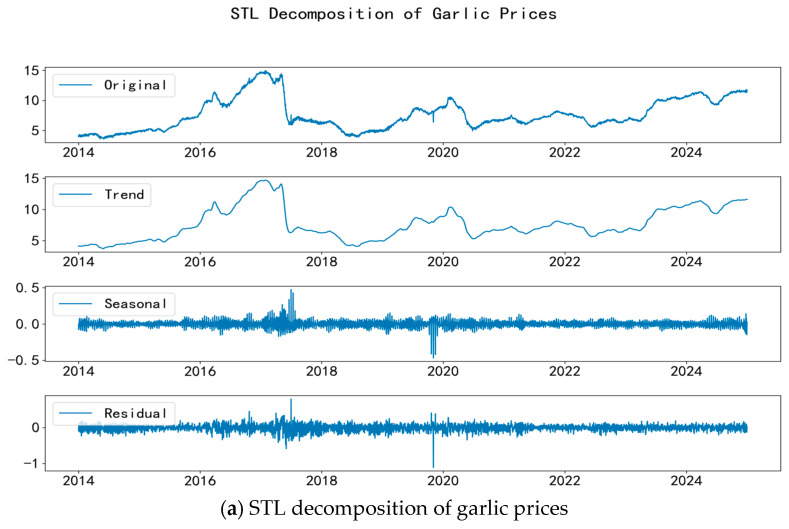

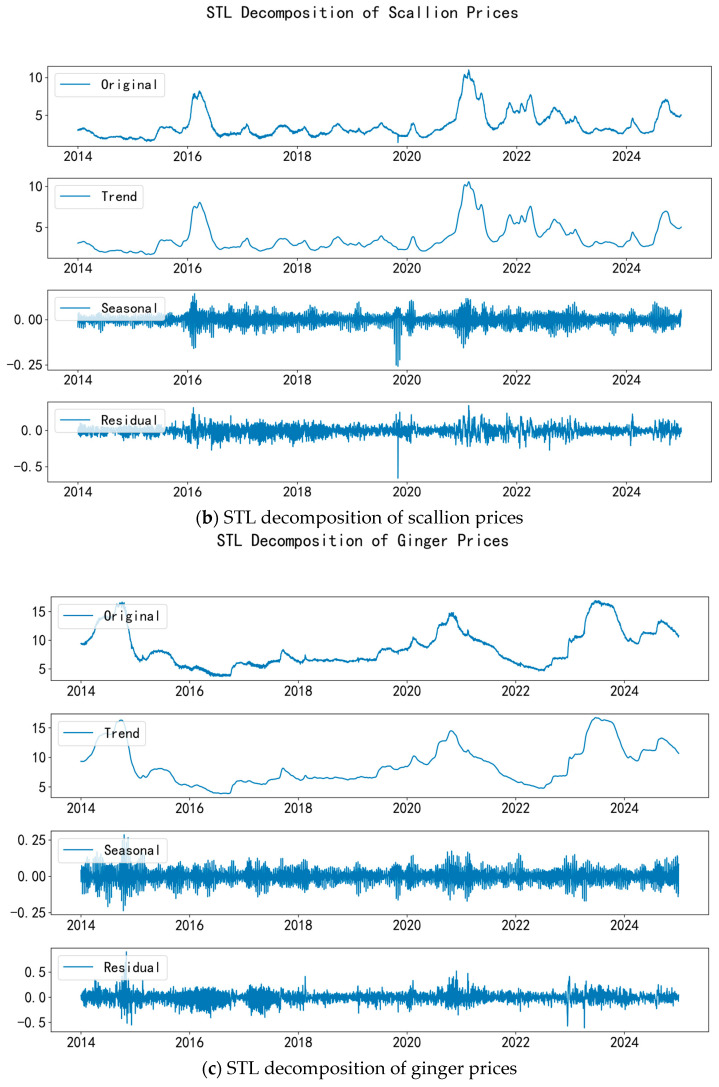
STL decomposition results for garlic, scallion, and ginger prices.

In this framework, STL is not used only as a smoothing tool. It reorganizes the price sequence into components with clearer temporal meanings. The trend component captures slow structural movement, the seasonal component represents recurring production and market cycles, and the residual component retains short-term irregular changes. This treatment is consistent with previous studies showing that STL-based hybrid models can improve forecasting performance for agricultural and nonlinear time-series data [22,24]. The STL decomposition results for garlic, scallion, and ginger prices are shown in Figure 5.

#### 2.5.3. EMA-Based Multi-Scale Temporal Representation

Although STL separates the raw price series into structural components, the decomposed signals may still contain heterogeneous temporal dependence. Short-term shocks, medium-term market adjustment, and longer-term structural movement can coexist in the input sequence. Treating all historical observations equally may reduce the model’s ability to identify the most relevant temporal information.

To address this issue, the Efficient Multi-Scale Attention (EMA) module was introduced to refine temporal representations [27]. Let $q_{t}$ denote the hidden representation derived from the structured input $z_{t}$. EMA produces a weighted temporal representation:

(8) $${\tilde{q}}_{t}=\sum_{i=0}^{L-1}{\alpha_{i}q}_{t-i}$$

where $\alpha_{i}$ denotes the adaptive weight assigned to the *i*-th historical representation within the temporal window.

The attention weights are computed as

(9) $$\alpha_{i}=\frac{\exp(e_{i})}{\sum_{j=0}^{L-1}\exp(e_{j})}$$

where $e_{i}$ is the attention score generated from trainable parameters.

The EMA module receives the structured input sequence after STL decomposition and feature concatenation. For each sliding-window sample, the input tensor can be denoted as X_t_ ∈ R^L×d^, where L is the input window length, set to 30 in this study, and d is the number of decomposed price components and explanatory variables. The EMA module first projects the input sequence into hidden temporal representations and then assigns adaptive weights to different temporal positions within the window. The weighted representation is subsequently passed to the LSTM predictor for sequence learning and final price estimation. In this way, EMA acts as a temporal feature-refinement module rather than as an independent forecasting model. This design allows the model to adjust the relative importance of historical information according to the current forecasting context, which is useful for specialty fresh food commodity prices because some fluctuations are short-lived, whereas others reflect more persistent market adjustment.

#### 2.5.4. Economically Motivated Physics-Informed Trajectory-Regularized Constraint

A purely data-driven sequence model may fit historical prices well, but it can still generate locally erratic forecast paths during volatile market periods. To improve trajectory stability and economic plausibility, this study introduces an economically motivated physics-informed trajectory-regularized constraint into the LSTM predictor. The proposed PILSTM module draws on the general physics-informed learning idea of combining a data-fitting term with a residual-based structural constraint. However, unlike conventional PINNs that use explicit governing physical equations, the residual term in this study is constructed from the local curvature of the predicted price trajectory. It is motivated by price-adjustment inertia, market-adjustment continuity, and the local smoothness of short-term wholesale price movements. Therefore, the term “physics-informed” is used here in a constraint-guided sense and does not imply that food commodity prices follow a strict physical law or a complete structural market equation. Instead, the constraint provides an economically motivated trajectory-regularization term that penalizes unsupported local oscillations while allowing the model to respond to genuine market fluctuations captured by historical prices and explanatory variables [28,29,30,31,32].

The term “physics-informed” is used here in a constraint-guided sense. The residual constraint is not derived from an explicit physical governing equation. Instead, it follows the broader physics-informed learning principle of combining a data-fitting loss with a residual-based structural constraint. For short-term wholesale food prices, abrupt high-curvature forecast paths are often economically implausible unless they are supported by corresponding market signals. The second-order trajectory residual therefore serves as a regularization prior motivated by price-adjustment inertia, market-adjustment continuity, transaction frictions, delayed information diffusion, and local smoothness in short-term market adjustment. This constraint penalizes unsupported local oscillations while preserving the model’s ability to respond to genuine price changes reflected in historical prices and multi-source explanatory variables. Thus, the proposed PILSTM should be understood as a trajectory-regularized LSTM inspired by physics-informed learning, rather than as a conventional PINN based on strict governing physical equations.

For an ordered predicted price sequence, the local curvature of the forecast trajectory can be approximated by the second-order difference:

(10) $$r_{t}={\hat{y}}_{t+1}-2{\hat{y}}_{t}+{\hat{y}}_{t-1},\;t=2,\ldots ,N-1$$

where $r_{t}$ denotes the local curvature of the predicted price trajectory at time t, and N denotes the length of the ordered predicted sequence. A larger absolute value of $r_{t}$ indicates a sharper local reversal or oscillation in the forecast path.

Based on this curvature term, the physics-informed trajectory-regularization loss is defined as:

(11) $$L_{\mathrm{traj}}=\frac{1}{N-2}\sum_{t=2}^{N-1}r_{t}^{2}$$

The data-fitting loss is calculated using the mean squared error:

(12) $$L_{\mathrm{data}}=\frac{1}{N}\sum_{t=1}^{N}{\left(y_{t}-{\hat{y}}_{t}\right)}^{2}$$

The final training objective is:

(13) $$L=L_{\mathrm{data}}+\lambda L_{\mathrm{traj}}$$

where λ controls the strength of the trajectory-regularization constraint. In the experiments, λ was set to 0.4 according to validation-set performance during model-configuration selection. This constraint does not force the predicted path to become artificially flat or overly smoothed. Instead, it penalizes excessive local curvature and economically unsupported short-term oscillations in the predicted trajectory. In this way, the model is encouraged to fit observed price changes while maintaining a stable and economically plausible forecast path under volatile market conditions.

#### 2.5.5. LSTM-Based Sequence Learning

**Figure 6:**
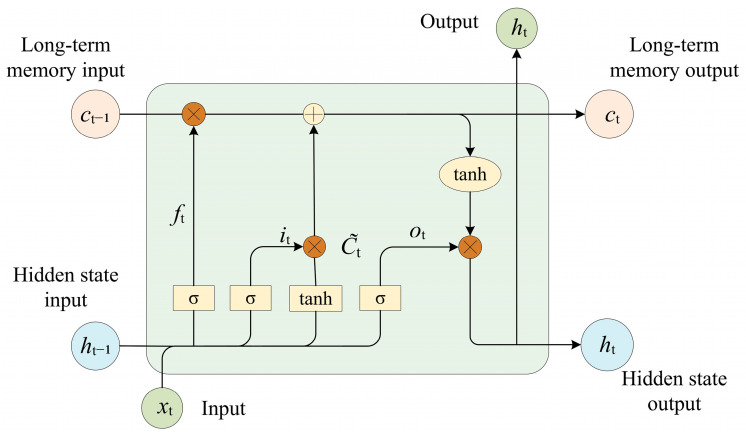
Computational structure of the LSTM unit embedded in the proposed STL–ETO–EMA–PILSTM framework.

The LSTM unit was used as the sequence-learning core of the proposed framework because it can capture nonlinear temporal dependence and retain useful historical information through its gating structure [15,17,33]. Figure 6 illustrates the computational structure of the LSTM unit embedded in the proposed framework.

Given the EMA-refined representation ${\tilde{q}}_{t}$, the LSTM updates its hidden state as:

(14) $$h_{t}=\mathrm{LSTM}({\tilde{q}}_{t},h_{t-1},c_{t-1})$$

where $h_{t}$ denotes the hidden state and $c_{t-1}$ denotes the previous cell state. The final prediction is obtained through a linear output layer:

(15) $${\hat{y}}_{t+1}=W_{y}h_{t}+b_{y}$$

In the proposed framework, LSTM does not operate as an isolated predictor. Its input is first reorganized by STL and refined by EMA, while its predicted path is constrained by the physics-informed trajectory-regularized constraint. The final forecast is therefore shaped by structural decomposition, multi-scale temporal representation, nonlinear sequence learning, and economically motivated trajectory control.

#### 2.5.6. ETO-Based Offline Model-Configuration Selection

The performance of the proposed STL–ETO–EMA–PILSTM framework depends on several interacting implementation settings, including the sequence-learning structure, optimizer configuration, training batch size, and trajectory-regularization strength. Manual tuning can be inefficient because these settings jointly affect forecasting accuracy and trajectory stability. Exponential-Trigonometric Optimization (ETO) was therefore adopted as a lightweight offline model-configuration search strategy before final model training [34].

In this study, ETO was used only before final model training. It was used to identify a suitable configuration for the coupled STL–ETO–EMA–PILSTM framework and did not enter the forward prediction process. Each candidate solution in the ETO population was encoded as a four-dimensional vector:

(16) $$X_{i}=\left[x_{i,1},x_{i,2},x_{i,3},x_{i,4}\right],i=1,2,\ldots ,N;D=4.$$

where Xi denotes the i-th candidate model configuration, xi,1, xi,2, xi,3, and xi,4 denote the encoded configuration variables, N denotes the population size, and D denotes the search dimension. The encoded values were mapped to feasible implementation settings during model training. This compact representation describes the ETO search space used in this study without implying that all model settings were independently optimized.

The quality of each candidate solution was evaluated using the validation error:

(17) $$\mathrm{Fitness}(X_{i})={\mathrm{RMSE}}_{\mathrm{val}}(X_{i})$$

(18) $${\mathrm{RMSE}}_{\mathrm{val}}\left(X_{i}\right)=\sqrt{\frac{1}{n_{\mathrm{val}}}\sum_{j=1}^{n_{\mathrm{val}}}{\left(y_{j}-{\hat{y}}_{j}\left(X_{i}\right)\right)}^{2}}$$

where ${\mathrm{RMSE}}_{\mathrm{val}}(X_{i})$ denotes the root mean squared error on the validation subset corresponding to candidate configuration X_i_.nval denotes the number of validation samples, y_j_ denotes the observed value of the j-th validation sample, and yhatj(X_i_) denotes the predicted value generated under candidate configuration X_i_. The candidate with the lowest validation RMSE was selected for final model training.

The ETO search dimension was set to D = 4, the population size was set to N = 2, and the maximum number of iterations was set to T = 5. Therefore, the total number of candidate-configuration evaluations was:

(19) M=N×T=2×5=10.

Each candidate configuration was trained on the training subset and evaluated on the validation subset using RMSE as the objective function. The search was stopped when the maximum number of iterations was reached. In this study, ETO was used as a lightweight offline model-configuration search procedure under a fixed computational budget. The population size was set to N = 2, and the maximum number of iterations was set to T = 5, giving M = N×T = 10 candidate-configuration evaluations. This setting should not be interpreted as an exhaustive global hyperparameter optimization process or as a guarantee of globally optimal or near-optimal hyperparameters. Instead, it was used to identify an empirically suitable configuration under the constraints of computational cost, validation-set stability, and reproducibility. The selected configuration was further evaluated through out-of-sample benchmark comparison, ablation analysis, multi-horizon forecasting, and Diebold–Mariano tests.

After ETO-based configuration selection, the final predictor consisted of an LSTM layer with 64 hidden units, an EMA module, and a Dense output layer. The model was trained using the Adam optimizer with a learning rate of 0.0001 for 30 epochs and a batch size of 16. The trajectory-regularization coefficient was set to λ = 0.4. The testing set was not used during configuration selection, preprocessing parameter estimation, or model selection. After the final configuration was fixed, the prediction stage was carried out by the STL, EMA, and trajectory-regularized LSTM components. Therefore, ETO should be understood as part of the offline training and configuration-selection pipeline, not as an additional inference module or as evidence of guaranteed global optimality.

### 2.6. Experimental Environment and Implementation

All experiments were conducted on a high-performance computing server equipped with an AMD EPYC 9754 processor (128 cores, 2.25 GHz), an NVIDIA RTX 4060 GPU with 8 GB memory, and 384 GB DDR5 RAM. The operating system was Ubuntu 20.04 LTS.

The proposed framework and all benchmark models were implemented in Python 3.8. PyTorch 1.9.0 was used for neural-network construction and training. NumPy 1.21.0 and Pandas 1.3.0 were used for data processing, while Matplotlib 3.4.3 and Seaborn 0.11.2 were used for visualization. All models were trained and evaluated under the same chronological train–test split and the same one-step-ahead forecasting setting.

### 2.7. Baseline Models and Hyperparameter Settings

To evaluate the forecasting performance of the proposed framework, the revised manuscript used nine benchmark models, including six conventional machine learning baselines and three additional time-series forecasting baselines. The conventional baselines included Support Vector Machine (SVM), Extreme Gradient Boosting (XGBoost), Lasso Regression, Random Forest, Gradient Boosting, and LightGBM. To strengthen the comparison with persistence-based and recent deep time-series forecasting methods, Naive persistence, Transformer Encoder, and PatchTST were further included. All benchmark models used the same input variables, sliding-window setting, chronological train–test split, and evaluation metrics as the proposed model.

The implementation settings and ETO search budget of the proposed model are shown in Table 2. The settings of the benchmark models are shown in Table 3. All benchmark models used the same input variables, sliding-window setting, chronological train–test split, and evaluation metrics as the proposed model.

The naive persistence model was included as a strong short-term reference because daily agricultural prices often exhibit temporal persistence. Transformer Encoder and PatchTST were included as representative modern deep time-series forecasting models. Because the dataset contains a limited number of daily observations for each commodity and the forecasting task is dominated by short-term price persistence, these models were used as additional reference architectures rather than extensively tuned large-scale forecasting systems.

### 2.8. Evaluation Metrics

Four standard regression metrics were used to evaluate forecasting performance: Mean Absolute Error (MAE), Mean Squared Error (MSE), Root Mean Squared Error (RMSE), and the coefficient of determination ($R^{2}$). MAE measures the average absolute deviation between observed and predicted prices. MSE and RMSE assign greater weight to larger errors, while $R^{2}$ measures the goodness of fit between observed and predicted series.

(20) $$\mathrm{MAE}=\frac{1}{N}\sum_{i=1}^{N}\left|y_{i}-{\hat{y}}_{i}\right|$$

(21) $$\mathrm{MSE}=\frac{1}{N}\sum_{i=1}^{N}{\left(y_{i}-{\hat{y}}_{i}\right)}^{2}$$

(22) $$\mathrm{RMSE}=\sqrt{\frac{1}{N}\sum_{i=1}^{N}{\left(y_{i}-{\hat{y}}_{i}\right)}^{2}}$$

(23) $$R^{2}=1-\frac{\sum_{i=1}^{N}(y_{i}-{\hat{y}}_{i}{)}^{2}}{\sum_{i=1}^{N}(y_{i}-\bar{y}{)}^{2}}$$

where $y_{i}$ denotes the observed price, ${\hat{y}}_{i}$ denotes the predicted price, $\bar{y}$ denotes the mean observed price, and N denotes the number of testing samples. Lower MAE, MSE, and RMSE values indicate better forecasting accuracy, while a higher $R^{2}$ indicates stronger explanatory power.

### 2.9. Temporal Alignment and Leakage Prevention

To reduce the risk of temporal leakage, all variables were aligned according to the information that would have been available before the forecasting date. For a target $value(y_{t+h})$, the input window contained only historical prices and explanatory variables observed or officially available no later than time (t). No future target values or future explanatory variables were included in the model inputs.

Daily variables, including exchange rates, crude-oil prices, temperature, and online attention indicators, were lagged when their same-day values would not have been available before prediction. Monthly indicators, including CPI, M2, export value, and export volume, were shifted according to reporting availability and carried forward only after official release. Annual planting area and production variables were represented by the most recently available annual statistics, rather than by future annual totals. This setting was used to approximate a realistic forecasting scenario rather than a retrospective fitting task.

The chronological split was applied before model fitting, hyperparameter selection, and performance evaluation. Normalization parameters were estimated only from the training subset and then applied unchanged to the validation and testing subsets. The testing subset was not used for ETO-based hyperparameter optimization, early stopping, model selection, or preprocessing parameter estimation. STL decomposition was implemented in a training-only or rolling manner so that future test-period observations were not used to construct decomposition-based inputs for earlier forecasts. These procedures ensured that each prediction was generated using only information available at the corresponding forecasting time.

## 3. Results

### 3.1. Forecasting Performance of the Proposed Framework

The forecasting performance of the proposed STL–ETO–EMA–PILSTM framework was evaluated on garlic, scallion, and ginger price series under the one-step-ahead forecasting setting. Table 4 reports the results on the testing set. The framework produced low prediction errors for all three specialty fresh food commodities, with $R^{2}$ values above 0.996. Among the three commodities, scallion obtained the lowest MAE and RMSE, followed by garlic, while ginger showed larger errors because of its stronger local volatility and less regular short-term movement.

The high one-step R^2^ values should be interpreted together with the additional validation results rather than as standalone evidence of forecasting reliability. In daily wholesale price forecasting, adjacent observations are highly autocorrelated, and the naïve persistence baseline also produced high one-step R^2^ values. This suggests that part of the high R^2^ is related to the temporal persistence of daily price series. However, the proposed framework still achieved lower MAE and RMSE than the naïve baseline. In addition, the rolling-origin validation showed that model performance varied across different market regimes, and the direct multi-horizon experiment showed clear performance degradation as the forecasting horizon increased, especially at the 14-day horizon. These results indicate that the model did not obtain uniformly high performance under all validation settings and reduce the likelihood that the reported one-step results were caused by temporal leakage.

The cross-commodity differences are consistent with the volatility patterns observed in the original price series. Garlic and scallions have relatively clearer short-term regularity, whereas ginger contains stronger irregular fluctuation. Even for ginger, the proposed framework retained a high level of fit and stable trajectory tracking, suggesting that the model can handle heterogeneous price movements rather than fitting only one commodity-specific pattern.

**Figure 7:**
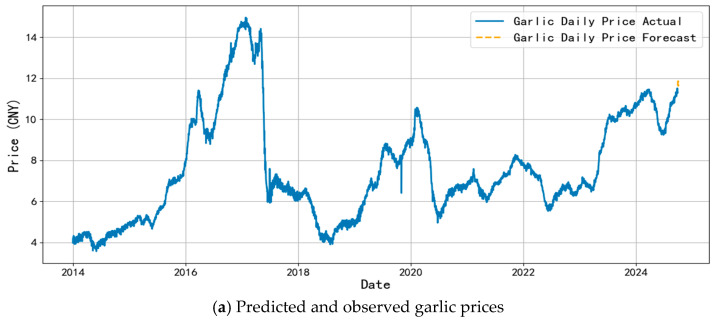

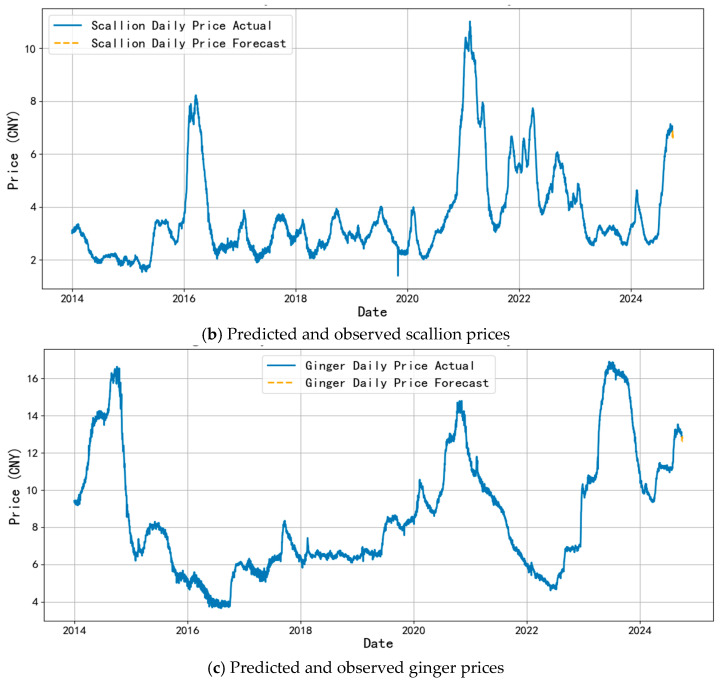
Observed and predicted prices for garlic, scallion, and ginger using the proposed STL–ETO–EMA–PILSTM framework.

In Figure 7, the predicted curves closely follow the main movements of the observed price series. The framework captures the dominant trajectories and major turning points without producing excessive local oscillations. This pattern is relevant to food market monitoring because useful forecasts should reduce pointwise error while maintaining stable and economically plausible trajectories, thereby supporting the early identification of abnormal price movements in fresh food markets.

The high R^2^ values should not be interpreted as evidence that food commodity price forecasting is inherently simple. They reflect the contribution of the full framework. STL decomposition separates mixed temporal components, EMA refines information across time scales, PILSTM introduces physics-informed trajectory regularization, and ETO supports offline model-configuration selection. These components jointly support forecasting accuracy, trajectory stability, and robustness under heterogeneous market conditions.

### 3.2. Benchmark Comparison

**Figure 8:**
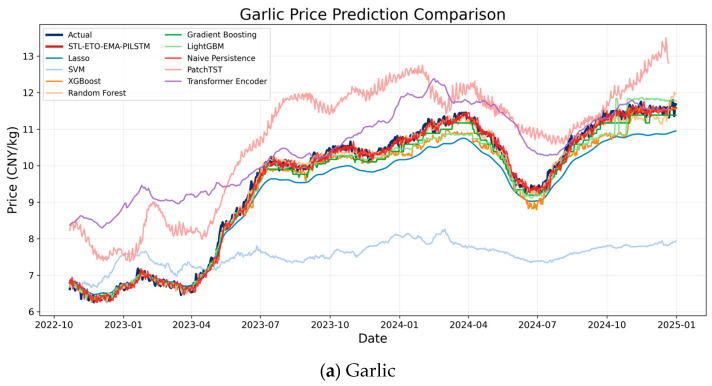

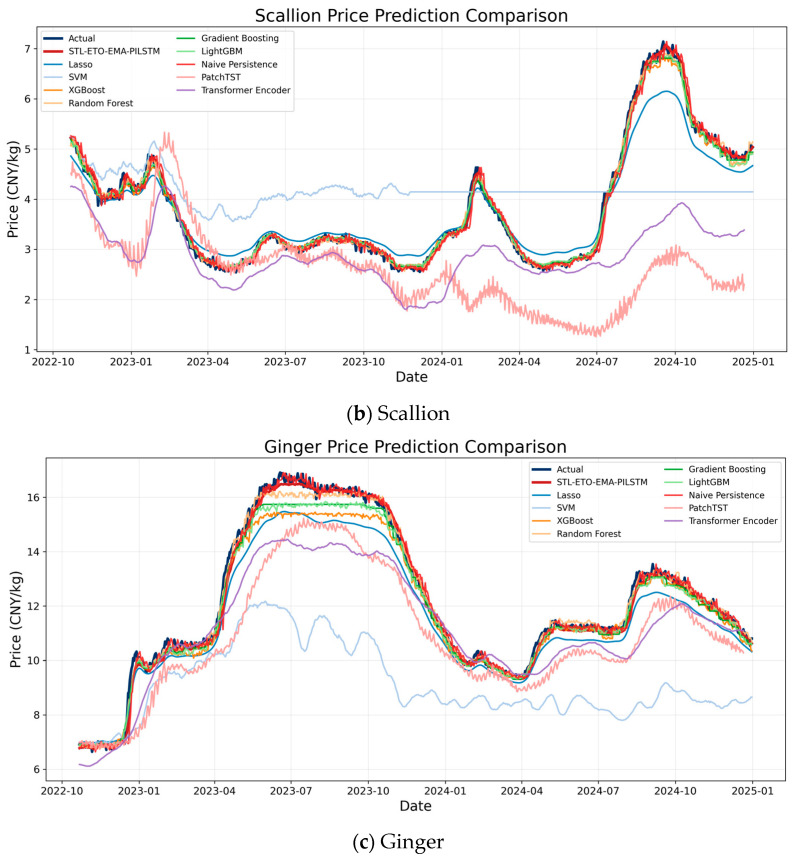
Benchmark comparison of observed and predicted prices for garlic, scallion, and ginger.

To evaluate the effectiveness of the proposed framework, nine benchmark models were used for comparison, including SVM, XGBoost, Lasso, Random Forest, Gradient Boosting, LightGBM, Naive persistence, Transformer Encoder, and PatchTST. All benchmark models used the same input variables, sliding-window setting, chronological train–test split, and evaluation metrics as the proposed model. This setting ensured that the comparison focused on model performance rather than differences in data preparation. The results for garlic, scallion, and ginger are reported in Table 5, Table 6 and Table 7, and the corresponding prediction curves are shown in Figure 8.

#### 3.2.1. Garlic Price Forecasting

Table 5 presents the benchmark comparison for garlic price forecasting. The proposed framework achieved the best performance across all four evaluation metrics. Among the benchmark models, Random Forest and Gradient Boosting were relatively competitive, but their errors remained higher than those of the proposed framework. SVM and Lasso performed poorly, indicating that static regression or kernel-based fitting alone is insufficient for capturing garlic price dynamics.

Garlic prices are relatively more regular than ginger prices, but they still contain mixed structural movement, seasonal variation, and nonlinear short-term fluctuation. The proposed framework performed better because it does not rely on a single modeling mechanism. STL separates structural components, EMA refines temporal information across different scales, and PILSTM constrains the predicted trajectory through physics-informed regularization. This combination helps the model reconstruct garlic price dynamics more accurately than the benchmark models.

#### 3.2.2. Scallion Price Forecasting

Table 6 reports the benchmark results for scallion price forecasting. The framework ranked first across all metrics. XGBoost, Random Forest, Gradient Boosting, and LightGBM performed better than SVM and Lasso, but their errors were still higher than those of the proposed framework.

Scallion prices are closely linked to short-term domestic supply and circulation conditions. The performance gap between the proposed framework and the benchmark models suggests that scallion price forecasting requires temporal structure modeling rather than static feature fitting alone. The proposed framework remains stronger because it separates mixed temporal components before sequence learning and reduces unstable local fitting through trajectory regularization.

#### 3.2.3. Ginger Price Forecasting

Table 7 presents the benchmark comparison for ginger price forecasting. Ginger was the most difficult commodity among the three because its price path showed stronger irregularity. Even in this case, the proposed model achieved the lowest MAE, MSE, and RMSE, as well as the highest R^2^. Random Forest was the closest benchmark, but its error values remained higher than those of the proposed framework.

The ginger results highlight the value of trajectory stability under irregular market conditions. Simpler models struggled more clearly when local disturbance increased. In contrast, the proposed framework reduced the interference of mixed temporal components through STL, refined temporal information through EMA, and discouraged implausible local oscillations through physics-informed trajectory regularization. This made the forecast less sensitive to short-term noise while preserving the main direction of price movement.

Figure 8 supports the quantitative results in Table 5, Table 6 and Table 7. Across the three commodities, the STL–ETO–EMA–PILSTM model remained closer to the observed price trajectories than the benchmark models. This advantage was especially clear near local extrema and directional changes, where forecast stability is more difficult to maintain but important for identifying abnormal price movements in fresh food markets. Lasso showed underfitting, SVM responded slowly to abrupt changes, and the tree-based ensemble models were more competitive but still showed larger deviations during short-term fluctuations.

The proposed framework did not improve performance by generating a sharper or more erratic curve. It followed the dominant price path while avoiding unsupported local reversals. This behavior is consistent with the role of physics-informed trajectory regularization and explains why the model performed well in both numerical accuracy and forecast-path stability.

### 3.3. Multi-Horizon Forecasting Performance

The multi-step forecasting results show that forecasting performance generally becomes less stable as the prediction horizon becomes longer, especially when the horizon extends to t + 14. Garlic maintains relatively stable short-term performance at t + 3 and t + 7, but its performance declines substantially at t + 14. Scallion shows the strongest horizon sensitivity, reflecting abrupt short-term market fluctuations during the testing period. Ginger remains relatively stable at t + 3 and t + 7, but also shows clear degradation at t + 14. These results support the positioning of the proposed framework as a short-term market-monitoring tool rather than a long-horizon structural projection model. Table 8 summarizes the detailed multi-step forecasting performance of the proposed framework at the 3-day, 7-day, and 14-day horizons.

### 3.4. Statistical Significance Testing

To examine whether the forecasting improvements were statistically meaningful, paired Diebold–Mariano tests were conducted between the proposed model and the benchmark models. The tests were based on paired forecast errors from the corresponding testing periods and were performed using both squared-error and absolute-error loss functions. A *p*-value below 0.05 indicates that the difference in paired forecast errors is statistically significant at the 5% level.

Table 9 summarizes the Diebold–Mariano test results across three commodities, nine benchmark models, four forecasting settings, and two loss functions. Overall, 190 out of 216 paired comparisons were statistically significant at the 5% level, indicating that the improvement of the proposed framework is not limited to raw metric differences in MAE, MSE, RMSE, and R^2^. The significance pattern was stronger under shorter forecasting horizons. In the one-step-ahead, 3-day, 7-day, and 14-day settings, 52/54, 50/54, 52/54, and 36/54 comparisons were significant, respectively.

This horizon-related pattern is consistent with the multi-horizon forecasting results, where predictive performance generally became less stable as the forecasting horizon became longer. The DM test results therefore support the short-term advantage of the proposed framework, while also showing that its relative advantage becomes less stable under longer-horizon forecasting. The non-significant cases were reported rather than omitted. In the one-step-ahead setting, the garlic–Lasso comparison under squared-error loss and the scallion–PatchTST comparison under absolute-error loss were not significant at the 5% level. More non-significant cases appeared at the 14-day horizon, especially for ginger and for comparisons with several strong baseline models.

Therefore, the proposed framework is not described as statistically superior in every single comparison. Instead, the results indicate that it achieves statistically significant improvements in most paired comparisons, particularly under short-horizon food-market monitoring settings. A summary of the pairwise Diebold–Mariano test results is reported in Table 9.

### 3.5. Ablation Study

An ablation study was conducted to examine the contribution of the main components in the proposed framework. Since ETO is an offline hyperparameter search strategy rather than a forward propagation module, the ablation analysis focused on the components that directly affect feature representation and sequence prediction. Three variants were compared: plain LSTM, EMA–PINN–LSTM, and the full STL–ETO–EMA–PILSTM framework.

The plain LSTM served as a basic sequence-learning baseline without STL decomposition, EMA-based temporal refinement, or physics-informed trajectory regularization. EMA–PINN–LSTM denotes the variant that retains EMA-based temporal refinement and the physics-informed trajectory-regularized LSTM predictor but removes STL decomposition. The full STL–ETO–EMA–PILSTM framework further adds STL-based structural decomposition and ETO-based offline model-configuration selection. All variants used the same dataset, sliding-window setting, and chronological train–test split.

#### 3.5.1. Garlic Price Ablation Results

Table 10 reports the ablation results for garlic price forecasting. The full model performed best, followed by EMA–PINN–LSTM and plain LSTM. The large performance loss of plain LSTM shows that direct sequence learning alone is not sufficient for stable garlic price forecasting.

Adding EMA and PINN already improved the plain LSTM, indicating that temporal refinement and trajectory regularization contribute to the forecast. Adding STL decomposition produced a further gain, suggesting that structural separation of trend, seasonal movement, and residual disturbance helps stabilize garlic price prediction.

**Figure 9:**
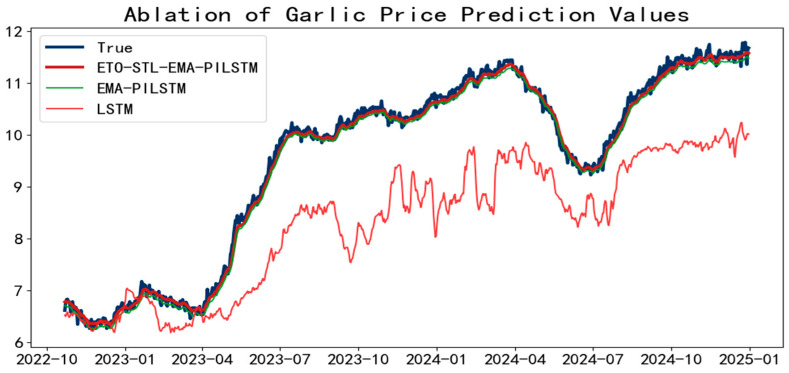
Ablation comparison for garlic price forecasting.

Figure 9 further supports the ablation results for garlic. The full STL–ETO–EMA–PILSTM model follows the observed price trajectory more closely and captures turning points with smaller deviations, indicating the value of combining decomposition, multi-scale representation, trajectory regularization, and hyperparameter optimization. EMA–PINN–LSTM remains competitive but shows larger local errors near extrema, while plain LSTM does not adequately reproduce the overall trajectory.

#### 3.5.2. Scallion Price Ablation Results

Table 11 reports the ablation results for scallion price forecasting. The full model again achieved the best performance. EMA–PINN–LSTM remained close to the full model, but removing STL still reduced accuracy and stability.

Scallion prices contain stronger short-term variability than garlic prices. The reduced EMA–PINN–LSTM model captured much of the useful structure, showing the value of multi-scale representation and trajectory regularization. The full model improved the result further by reducing the interference caused by mixed temporal components before sequence learning.

**Figure 10:**
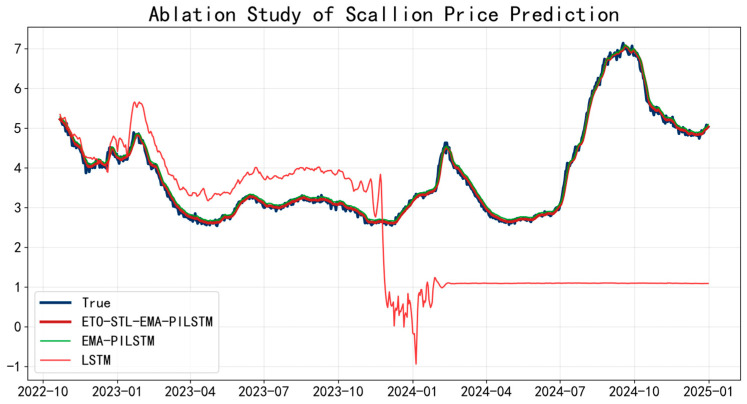
Ablation comparison for scallion price forecasting.

Figure 10 further supports the ablation results for scallion. The full STL–ETO–EMA–PILSTM model tracks the observed price trajectory more closely across the testing period, indicating the value of combining decomposition, multi-scale representation, trajectory regularization, and hyperparameter optimization. EMA–PINN–LSTM captures the main movement but shows larger deviations near local turning points, while plain LSTM produces less stable forecasts and does not adequately preserve the overall trajectory.

#### 3.5.3. Ginger Price Ablation Results

Table 12 reports the ablation results for ginger price forecasting. Ginger represents the most demanding case because its price movement is more irregular. The full model still performed best, EMA–PINN–LSTM ranked second, and plain LSTM showed a much weaker fit.

The ginger case confirms the importance of robustness under high volatility. EMA–PINN–LSTM already improved substantially over plain LSTM, indicating that multi-scale representation and trajectory regularization help stabilize the forecast. The full model improved the result further, showing that STL decomposition remains useful even when short-term fluctuations are strong.

**Figure 11:**
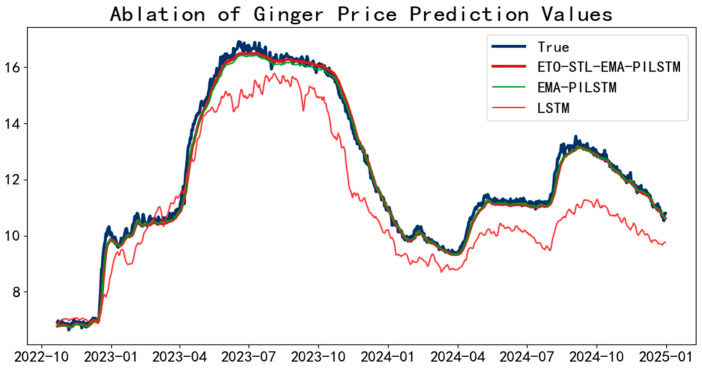
Ablation comparison for ginger price forecasting.

Figure 11 further supports the ablation results for ginger. The full STL–ETO–EMA–PILSTM model remains closest to the observed price trajectory. EMA–PINN–LSTM shows larger local deviations, while plain LSTM does not adequately track the main movement of the series. These results indicate that decomposition, temporal refinement, trajectory regularization, and offline hyperparameter search jointly improve forecasting robustness under irregular market conditions.

### 3.6. Interpretability and Market-Driver Analysis

To examine how the proposed framework forms its forecasts, feature-importance and interaction analyses were conducted on the trained STL–ETO–EMA–PILSTM model. This analysis was used to identify which market signals contributed most strongly to price prediction and whether the model captured commodity-specific driver structures.

#### 3.6.1. Feature Importance Analysis

**Figure 12:**
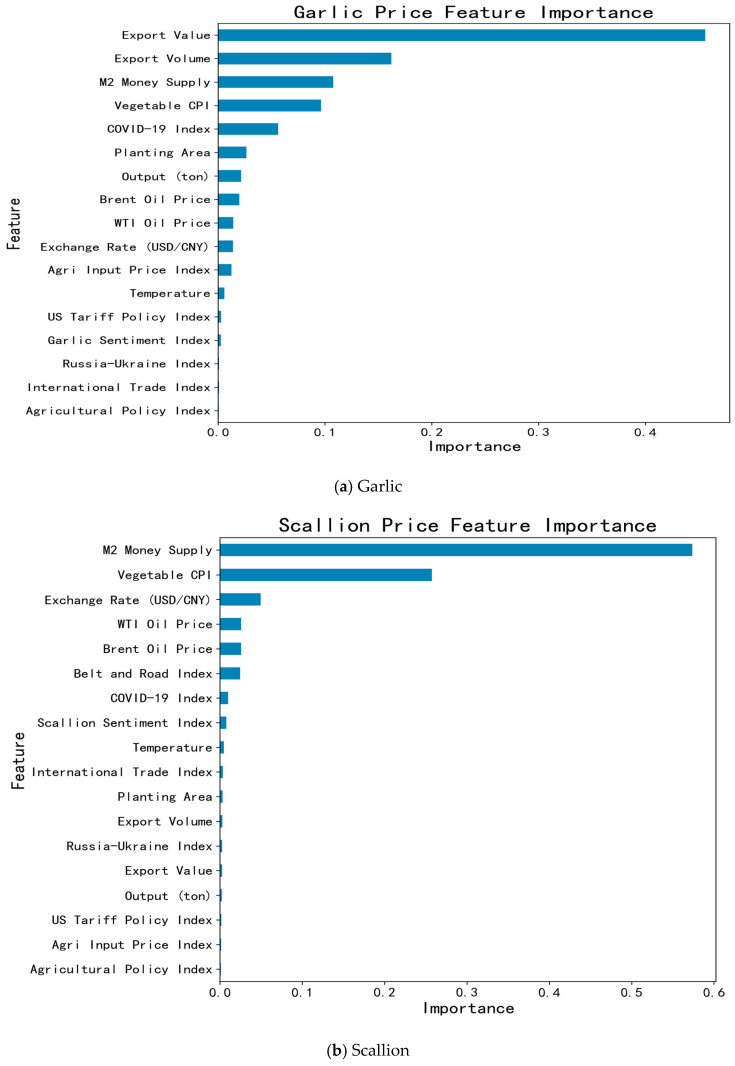

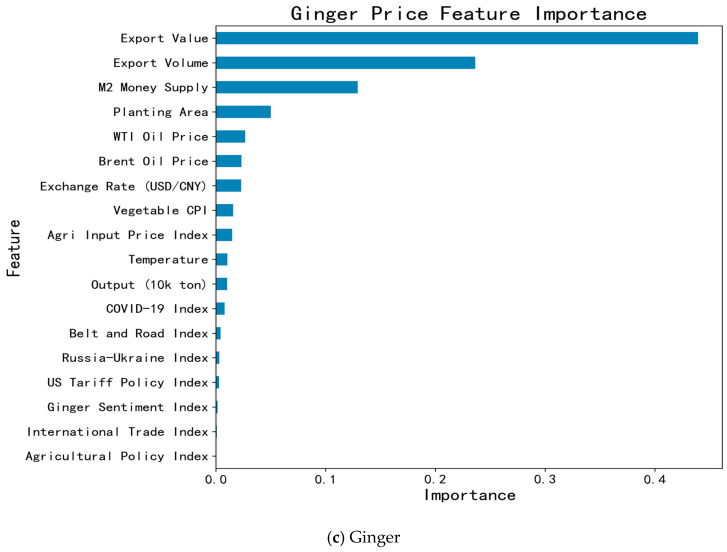
Permutation-based feature importance for garlic, scallion, and ginger price forecasting.

Before presenting the feature-importance results, the calculation method is clarified here. The feature-importance values shown in Figure 12 were derived from a permutation-based predictive-importance calculation on the trained STL–ETO–EMA–PILSTM model. For each feature, its values in the testing subset were perturbed while the remaining variables were kept unchanged. The increase in prediction error after perturbation was used as the importance score of that feature. A larger error increase indicates that the trained model depends more strongly on that feature for prediction. The importance scores were normalized within each commodity to make the relative contribution of variables easier to compare. These results should be interpreted as model-based predictive importance rather than SHAP values, attention weights, or causal effects.

Based on this procedure, Figure 12 shows that the relative contribution of variables differs across the three commodities. For garlic, export-related variables make the largest contribution, especially export value and export volume. This pattern points to a close association between garlic prices, external demand, and trade-side market conditions. Macroeconomic indicators, including M2 and the fresh vegetable CPI, also contribute to prediction, indicating that broader demand and liquidity conditions affect domestic garlic prices.

For scallion, the importance structure is different. Historical price information, domestic supply-related variables, temperature, fresh vegetable CPI, and M2 are more prominent than export variables. This is consistent with the nature of scallion as a more perishable fresh food commodity, whose price is strongly linked to short-term domestic supply and circulation conditions.

For ginger, feature importance is more evenly distributed. Export-related variables, macroeconomic indicators, online attention, and supply-side variables all contribute to prediction. This broader distribution is consistent with the stronger volatility observed in ginger prices and suggests that ginger price formation is shaped by multiple transmission channels rather than one dominant driver.

#### 3.6.2. Variable Interaction Analysis

**Figure 13:**
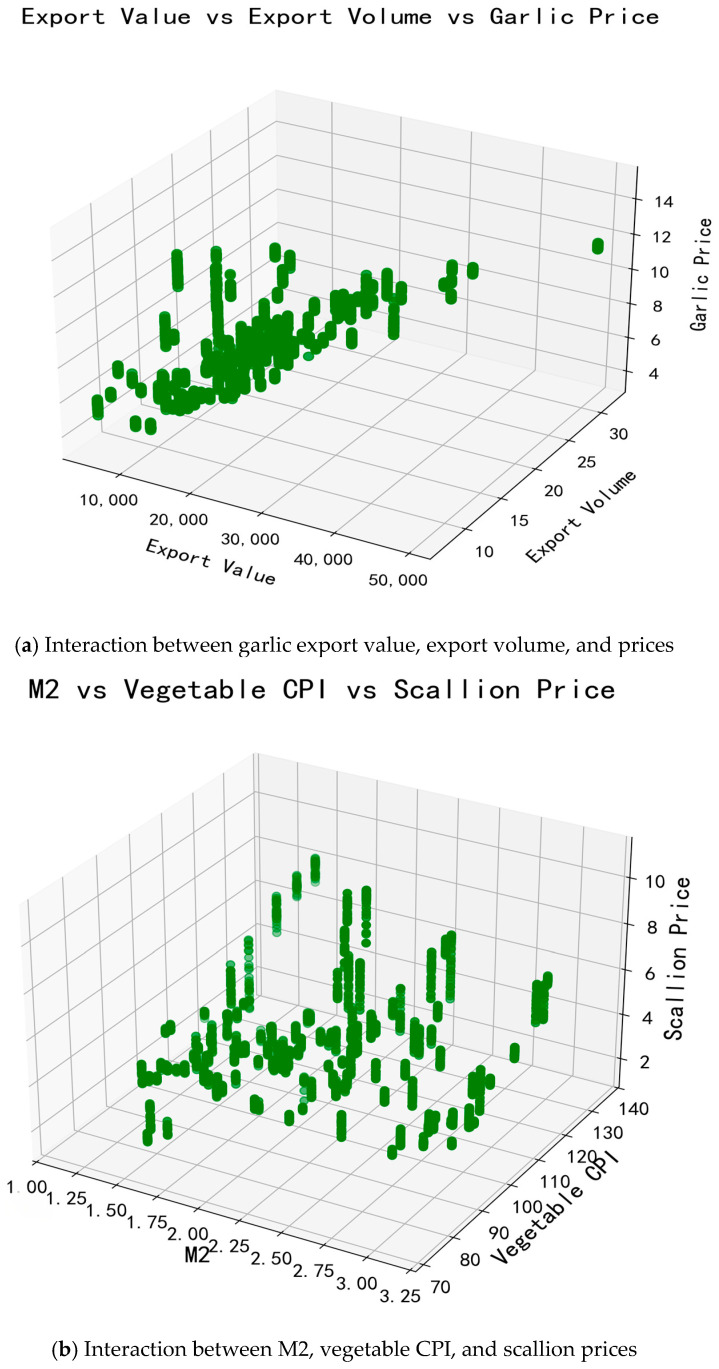

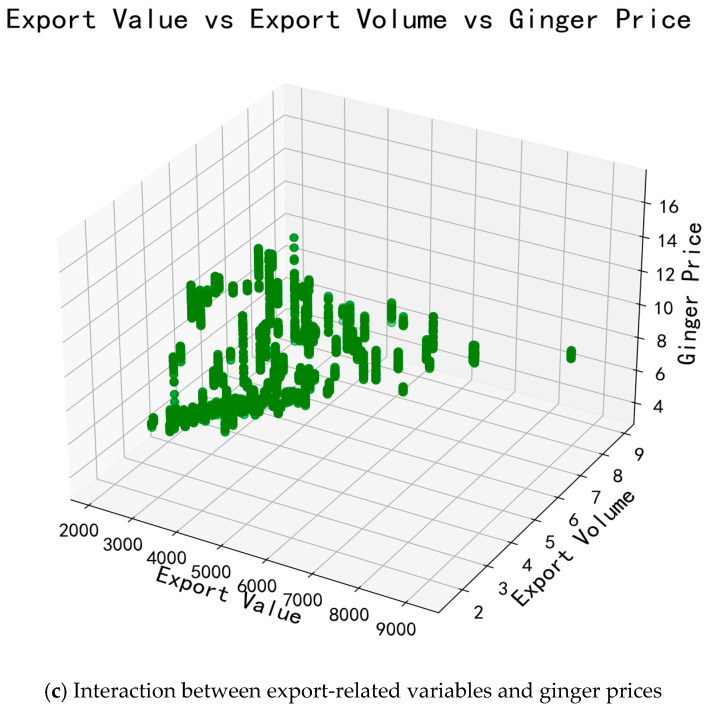
Variable-interaction analysis for garlic, scallion, and ginger price forecasting.

Figure 13 provides a more direct view of representative variable interactions. For garlic, export value and export volume show a jointly positive association with price. Stronger export demand may therefore tighten effective domestic supply and raise domestic prices. For scallion, M2 and the fresh vegetable CPI jointly affect prices through liquidity, demand, and consumer-price channels. For ginger, export-related variables remain important, but the response is less concentrated, reflecting a more mixed market mechanism.

These interaction patterns explain why a flexible forecasting framework is needed for heterogeneous specialty crops. Garlic shows a clearer trade-driven structure, scallion is more strongly linked to domestic supply and macroeconomic adjustment, and ginger reflects a broader combination of trade, macroeconomic, and production-side signals. The interpretability results support the use of multi-source inputs and show that the proposed framework can provide market-relevant information in addition to numerical forecasts.

### 3.7. Summary of Results

The results show that the proposed STL–ETO–EMA–PILSTM framework provides accurate and stable one-step-ahead forecasts for garlic, scallion, and ginger prices. The benchmark comparison demonstrates that the proposed model outperforms conventional machine learning models and additional time-series forecasting baselines across all three commodities. The ablation study confirms that the performance gain comes from the coordinated use of STL decomposition, EMA-based temporal refinement, physics-informed trajectory regularization, and ETO-based hyperparameter search rather than from a single component.

The framework also shows robustness across commodities with different volatility structures. Garlic and scallion are relatively easier to forecast, while ginger presents stronger irregular fluctuation. The proposed model remains effective in all three cases, suggesting that it is suitable for heterogeneous specialty fresh food commodity markets. Feature-importance and interaction analyses further show that the model captures commodity-specific market drivers, supporting its use for food commodity price forecasting, market stability monitoring, and short-term operational risk monitoring.

## 4. Discussion

The results of this study should be interpreted from the perspective of short-term fresh food market monitoring rather than only from the numerical improvement of forecasting errors. From a methodological perspective, recent optimized decomposition-based forecasting research on highly volatile crude-oil futures also provides useful context for using decomposition and hybrid learning to process energy-related market signals [35]. Recent food-price forecasting research has emphasized that price forecasts are not merely technical outputs but also public and market information that can support uncertainty communication, procurement adjustment, and short-term policy awareness during periods of rapid economic change [1]. This is particularly relevant for specialty fresh food commodities, whose prices may respond quickly to seasonal supply, circulation conditions, trade changes, energy costs, and online information diffusion. The proposed STL–ETO–EMA–PILSTM framework achieved lower errors than conventional machine learning models and additional time-series baselines, but its more important implication is that it provides stable short-term forecast trajectories and interpretable market signals for three heterogeneous commodity cases.

The high one-step-ahead R^2^ values should be understood in the context of daily wholesale price persistence. Agricultural and vegetable prices often show strong short-term autocorrelation, and recent studies have shown that deep-learning models can capture local temporal dependence in daily or high-frequency agricultural price series [2,25,36,37]. Therefore, high short-horizon fitting performance alone does not prove that a model is suitable for practical market monitoring. In this study, the added validation procedures, including leakage-prevention design, walk-forward validation, multi-horizon forecasting, and Diebold–Mariano tests, provide a more conservative basis for interpreting the results. The clear degradation of performance as the forecasting horizon increased indicates that the framework is better suited to short-term monitoring and early warning than to long-term structural price projection. This positioning is important because fresh food supply-chain decisions, such as procurement timing, replenishment, inventory adjustment, and abnormal price detection, are usually made over short operational horizons.

For the garlic data instance, the results suggest that garlic prices are more closely associated with export-related variables and trade-side market signals. This finding is consistent with the market characteristics of garlic as a specialty crop with concentrated production areas, relatively active interregional circulation, and stronger exposure to external demand than many leafy or highly perishable vegetables. From the perspective of market stability, the importance of export value and export volume means that garlic price monitoring should not rely only on domestic wholesale prices or production-side variables. When external demand strengthens, effective domestic supply may tighten, and traders’ expectations may change before price increases are fully reflected in wholesale markets. Conversely, weaker export demand may release more supply into domestic circulation and create downward price pressure. Therefore, for garlic, short-term market stability monitoring should pay attention to export demand, exchange-rate changes, international logistics costs, trade-policy signals, and online market sentiment. The proposed framework is useful in this case because it combines trade variables with domestic price inertia and then translates these mixed signals into a stable short-term forecast path.

For the scallion data instance, the results show a different market stability implication. Scallion prices were more closely linked with domestic supply conditions, temperature, fresh vegetable CPI, monetary conditions, and information-related variables. This pattern is reasonable because scallion is more perishable, more dependent on local circulation, and more sensitive to short-term supply disruption than garlic. Recent studies on vegetable price forecasting also show that daily vegetable prices contain strong short-term fluctuations and that LSTM-based or attention-enhanced models can support short-horizon prediction in vegetable markets. The interaction between M2 and fresh vegetable CPI in this study further suggests that scallion price changes are not only a production-side issue, but also reflect broader demand, liquidity, and consumer-price conditions. For market stability, this means that scallion monitoring should focus on domestic circulation pressure, weather-related supply disruption, short-term demand recovery, and consumer-price signals. A stable one-step-ahead forecast can help wholesalers, retailers, and regulators judge whether an observed price increase is likely to be a temporary local fluctuation or part of a broader short-term market adjustment. Compared with garlic, scallion requires faster operational response because its limited storability reduces the ability of inventory to buffer supply shocks.

For the ginger data instance, the results indicate a more mixed and less concentrated driver structure. Ginger had larger forecasting errors than garlic and scallion, and its feature-importance pattern involved trade, macroeconomic, supply-side, and information-related variables. This suggests that ginger price formation is affected by several transmission channels at the same time, rather than by one dominant market factor. From a market stability perspective, the ginger case is important because mixed drivers make simple rule-based monitoring unreliable. A price increase may come from production-side contraction, export demand, online attention, macroeconomic adjustment, or a combination of these factors. Therefore, for ginger, the value of the proposed framework lies not only in reducing forecast error but also in helping identify which groups of variables are contributing more strongly to the predicted movement. This is useful for distinguishing ordinary seasonal adjustment from broader volatility risk.

The cross-commodity differences show that specialty fresh food commodities should not be treated as a uniform price system. Garlic, scallion, and ginger are all seasoning-type fresh food commodities, but their market stability mechanisms differ. Garlic shows stronger trade-side transmission, scallion is more sensitive to domestic circulation and macro-consumption signals, and ginger reflects a broader mixture of trade, supply, macroeconomic, and information-related factors. This finding supports the need for a common modeling framework with commodity-specific interpretation. A single linear model or a purely historical-price model may capture short-term persistence, but it cannot fully explain why different commodities respond differently to external variables. Recent studies also emphasize that incorporating exogenous variables, market indicators, and commodity-specific features can improve agricultural and vegetable price prediction [2,37]. The present results extend this idea by showing that the usefulness of exogenous variables depends on the commodity-specific market-transmission mechanism.

The roles of STL decomposition, EMA-based temporal representation, and trajectory regularization should also be discussed from the viewpoint of market monitoring. STL decomposition helps separate trend, seasonal, and residual components, reducing the burden on the sequence learner when price series contain mixed temporal patterns. EMA further helps the model assign different weights to recent and earlier information within the input window. The trajectory-regularized PILSTM component then discourages economically unsupported high-frequency reversals in the predicted path. This is important for fresh food market applications because an early-warning system should not produce unstable signals that frequently reverse without corresponding market evidence. The ablation results suggest that the performance gain comes from the coordinated use of these components rather than from one isolated module. In practical terms, the framework improves both point forecasting and forecast-path plausibility, which is more relevant for market stability monitoring than numerical accuracy alone.

The feature-importance and interaction analyses provide additional market information, but they should be interpreted carefully. The results reveal predictive associations between variables and prices, not causal effects. For example, the importance of export value for garlic does not mean that export changes alone mechanically determine garlic prices; rather, it indicates that export-related information improves short-term prediction within the model. Similarly, the interaction between M2 and fresh vegetable CPI for scallion should be understood as a predictive signal related to macroeconomic and consumer-price conditions, rather than as a causal monetary-policy conclusion. This distinction is important because food-market monitoring requires both useful signals and cautious interpretation. Recent work on agricultural price forecasting has also emphasized the need to combine prediction with interpretability so that forecasts can support decisions by farmers, traders, firms, and public agencies [1,25].

The inclusion of trade, crude-oil, policy, shock, and information-related variables is supported by recent evidence on global food-market instability. The COVID-19 pandemic, trade frictions, and the Russia–Ukraine conflict have affected food and agricultural markets through trade disruption, cost transmission, energy-market pressure, and expectation adjustment [1,38]. The Russia–Ukraine war increased food, fuel, and fertilizer prices and created new pressures for food security and developing-country markets [38]. Trade-policy announcements can also increase global food commodity price volatility, especially under tight stock conditions [39]. In addition, geopolitical risk, supply-chain pressure, oil-price shocks, and shipping costs can transmit to food inflation and broader price dynamics [40,41]. Although garlic, scallion, and ginger are not global staple grains, their domestic prices can still be affected indirectly through energy costs, transportation expenses, input costs, trade expectations, and market sentiment. This explains why a multi-source variable system is more appropriate than a price-only model for short-term specialty fresh food commodity monitoring.

For practical deployment, the proposed framework should be used as a decision-support tool rather than an automatic policy-making system. Its forecasts can help market participants identify abnormal short-term movements, adjust procurement timing, and prepare for possible price-risk episodes. However, operational reliability depends on timely updates of wholesale prices, online-attention indicators, macroeconomic variables, trade statistics, and production-related information. Monthly and annual variables must be aligned according to their actual release dates, and missing or delayed data should be flagged rather than silently filled in a deployed system. When extreme weather, logistics disruption, disease outbreaks, sudden policy changes, or geopolitical shocks occur, model outputs should be interpreted together with expert judgment, market reports, and scenario analysis. This is especially important for specialty fresh food commodities because local shocks can be amplified quickly when storability is limited and production areas are concentrated.

This study still has several limitations. First, the empirical application focuses on garlic, scallion, and ginger in China. Although these commodities provide a heterogeneous setting, the framework should be validated on more vegetables, fruits, livestock products, and regional markets before broader generalization. Second, the current task is mainly one-step-ahead forecasting. The multi-horizon results show that forecast uncertainty increases as the horizon becomes longer, so future work should develop interval forecasting, probabilistic warning, and scenario-based risk assessment. Third, the physics-informed component is a trajectory-residual constraint rather than a full structural market model. Future studies could incorporate supply–demand balance, inventory adjustment, price-spread transmission, logistics constraints, and regional market linkages. Fourth, the interpretability analysis identifies predictive associations, but causal mechanisms should be examined through econometric or policy-oriented designs. Finally, future work should integrate more detailed variables on fertilizer prices, transportation costs, cold-chain logistics, extreme weather, shipping disruption, and geopolitical risk so that the framework can better support food-market resilience monitoring under abnormal external shocks.

## 5. Conclusions

This study developed a trajectory-regularized physics-informed hybrid forecasting framework, STL–ETO–EMA–PILSTM, for short-term price forecasting and market stability monitoring of specialty fresh food commodities. Garlic, scallion, and ginger were selected as representative crops because they are closely related to daily food consumption and are characterized by seasonal production, geographically concentrated supply, limited storability, and high sensitivity to climate, trade, energy, and information-related shocks. The proposed framework integrates STL-based structural decomposition, EMA-based multi-scale temporal representation, a physics-informed trajectory-regularized LSTM module based on an economically motivated trajectory residual constraint, and ETO-based offline model-configuration selection.

The model produced accurate and stable one-step-ahead forecasts across all three commodities. On the testing set, the MAE values reached 0.0853, 0.0581, and 0.1409 for garlic, scallion, and ginger, respectively, with R^2^ values above 0.996. Compared with conventional machine learning models and additional time-series forecasting baselines, including naive persistence, Transformer Encoder, and PatchTST, the proposed model achieved lower prediction errors and better overall fitting performance. The ablation results further confirmed that STL decomposition, EMA-based temporal refinement, physics-informed trajectory regularization, and ETO-based hyperparameter search each contributed to improving forecasting accuracy and trajectory stability.

The interpretability analysis revealed clear differences in market-driver structures among the three commodities. Garlic prices were more closely associated with export demand and trade-related factors; scallion prices were more sensitive to domestic supply conditions and macroeconomic adjustment, while ginger prices reflected a broader interaction of trade, macroeconomic, information-related, and supply-side variables. These findings indicate that the framework supports numerical forecasting while helping identify crop-specific drivers behind price fluctuations.

For food-market monitoring, the framework provides a data-driven tool for tracking short-term price movements, identifying potential volatility risks, and supporting operational risk monitoring in fresh food supply chains. The results suggest that combining multi-source data with economically motivated trajectory-regularized physics-informed learning can improve predictive performance and practical interpretability in short-term market-monitoring scenarios. However, the framework should not be interpreted as a long-term supply-chain optimization or structural policy-simulation model. Future work should extend the framework to more food commodities, regional markets, and country-specific market environments. Such extensions should reconstruct the explanatory-variable system according to commodity characteristics, market institutions, data-release calendars, logistics conditions, and trade exposure. Longer forecasting horizons, interval prediction, abnormal fluctuation warning, and scenario-based analysis should also be further explored under extreme market disruptions. Future work should also examine whether larger ETO search budgets, repeated independent searches, or alternative configuration-selection methods can further improve the stability of the selected model configuration.

## Figures and Tables

**Table 1:** Multi-source variables used for specialty fresh food commodity price forecasting.

Factor Category	Variables Included
Historical price factors	Historical garlic prices; historical scallion prices; historical ginger prices
Agricultural supply factors	Average daily temperature in major producing areas; planting area; production volume; agricultural production cost index
Macroeconomic indicators	Fresh vegetable consumer price index (CPI); broad money supply (M2); exchange rate (USD/CNY)
International market factors	Export value (lagged variable); export volume (lagged variable); international crude oil prices
Policy factors	Belt and Road index, China–US tariff policy index; agricultural policy index
Shock and information factors	Russian–Ukraine conflict index; COVID-19 index; online public attention index

Note. Export value and export volume were incorporated as lagged variables to reduce the risk of target leakage. Monthly and annual indicators were aligned according to their reporting frequency and were carried forward only after official availability. Online-attention indicators were lagged by one day. Only information available before the forecasting date was used as model input.

**Table 2:** Implementation settings and ETO search budget of the proposed STL–ETO–EMA–PILSTM model.

Item	Value Used in This Study
Input window length	30 days
Final predictor structure	LSTM(64) + EMA + Dense(1)
Optimizer	Adam
Learning rate	0.0001
Training epochs	30
Batch size	16
Trajectory-regularization coefficient	λ = 0.4
ETO search dimension	D = 4
ETO population size	N = 2
Maximum ETO iterations	T = 5
Candidate-configuration evaluations	M = N × T = 10
Stopping criterion	Maximum number of iterations reached
Validation objective	RMSE on the validation subset

**Table 3:** Hyperparameter settings of baseline models.

Model	Hyperparameters
SVM	RBF kernel, C = 100, γ = 0.01
XGBoost	n_estimators = 200, max_depth = 6, learning_rate = 0.05
Random Forest	n_estimators = 200, max_depth = 10
Gradient Boosting	n_estimators = 200, learning_rate = 0.05
LightGBM	n_estimators = 200, max_depth = 6, learning_rate = 0.05
Lasso	α = 0.001
Naive persistence	Next-step prediction equals the most recent observed value; no trainable hyperparameters.
Transformer Encoder	30-day input window; direct one-step-ahead forecasting; trained and evaluated under the same chronological split as the proposed model
PatchTST	30-day input window; patch-based time-series forecasting baseline; trained and evaluated under the same chronological split as the proposed model

**Table 4:** Forecasting performance of the proposed STL–ETO–EMA–PILSTM framework.

Metric	Garlic	Scallion	Ginger
MAE	0.0853	0.0581	0.1409
MSE	0.0145	0.0062	0.0350
RMSE	0.1192	0.0792	0.1871
R^2^	0.9975	0.9978	0.9967

Note: Results correspond to one-step-ahead forecasting on the testing dataset.

**Table 5:** Benchmark comparison for garlic price forecasting.

Evaluation Metric	Proposed Model	SVM	XGBoost	Lasso	Random Forest	Gradient Boosting	LightGBM	Naive Persistence	PatchTST	Transformer Encoder
MAE	0.0853	2.2236	0.1461	0.4116	0.0979	0.1051	0.1437	0.1010	1.2069	0.9524
MSE	0.0145	6.3062	0.0363	0.2198	0.0163	0.0174	0.0416	0.0161	1.6994	1.5556
RMSE	0.1192	2.5112	0.1905	0.4688	0.1275	0.1319	0.2041	0.1268	1.3036	1.2472
R^2^	0.9975	−1.0425	0.9882	0.9288	0.9947	0.9944	0.9865	0.9948	0.4504	0.4969

**Table 6:** Benchmark comparison for scallion price forecasting.

Evaluation Metric	Proposed Model	SVM	XGBoost	Lasso	Random Forest	Gradient Boosting	LightGBM	Naive Persistence	PatchTST	Transformer Encoder
MAE	0.0581	1.0159	0.0763	0.2434	0.0629	0.0659	0.0709	0.0906	1.3163	0.9335
MSE	0.0062	1.4039	0.0103	0.0956	0.0070	0.0080	0.0087	0.0156	3.0928	1.6076
RMSE	0.0792	1.1849	0.1015	0.3092	0.0839	0.0896	0.0931	0.1247	1.7586	1.2679
R^2^	0.9978	0.0115	0.9927	0.9327	0.9950	0.9944	0.9939	0.9891	−1.1699	−0.1279

**Table 7:** Benchmark comparison for ginger price forecasting.

Evaluation Metric	Proposed Model	SVM	XGBoost	Lasso	Random Forest	Gradient Boosting	LightGBM	Naive Persistence	PatchTST	Transformer Encoder
MAE	0.1409	3.0146	0.3623	0.6340	0.1640	0.2497	0.2706	0.1553	1.2614	1.0392
MSE	0.0350	12.0309	0.2538	0.5392	0.0531	0.1189	0.1392	0.0501	2.2064	1.7175
RMSE	0.1871	3.4686	0.5038	0.7343	0.2305	0.3448	0.3731	0.2239	1.4854	1.3105
R^2^	0.9967	−0.6488	0.9652	0.9261	0.9927	0.9837	0.9809	0.9933	0.7050	0.7703

**Table 8:** Multi-step forecasting performance for 3-day, 7-day, and 14-day horizons.

Commodity	Horizon	MAE	MSE	RMSE	R^2^
Garlic	t + 3	0.3972	0.2330	0.4827	0.9246
Garlic	t + 7	0.3560	0.1938	0.4402	0.9370
Garlic	t + 14	1.0225	1.5001	1.2248	0.5074
Scallion	t + 3	1.5350	3.0818	1.7555	−1.1622
Scallion	t + 7	3.2318	13.6384	3.6930	−8.6000
Scallion	t + 14	3.3459	16.8629	4.1064	−10.8970
Ginger	t + 3	0.7484	0.9092	0.9535	0.8784
Ginger	t + 7	0.8339	1.1311	1.0635	0.8460
Ginger	t + 14	1.7557	4.1827	2.0452	0.4123

**Table 9:** Summary of Diebold–Mariano test results across forecasting settings.

Forecasting Setting	Number of Paired Comparisons	Significant Comparisons at 5%	Non-Significant Comparisons	Significant Ratio
One-step-ahead	54	52	2	96.30%
3-day horizon	54	50	4	92.59%
7-day horizon	54	52	2	96.30%
14-day horizon	54	36	18	66.67%
Total	216	190	26	87.96%

Note: Diebold–Mariano tests were conducted between the proposed framework and the nine benchmark models across three commodities, four forecasting settings, and two loss functions. Both squared-error and absolute-error loss functions were used. A *p*-value below 0.05 indicates statistical significance at the 5% level.

**Table 10:** Ablation results for garlic price forecasting.

Metric	Proposed Model	EMA-PINN-LSTM	LSTM
MAE	0.0853	0.1172	2.8734
MSE	0.0145	0.0252	11.0846
RMSE	0.1192	0.1589	3.3294
R^2^	0.9975	0.9962	−2.5848

**Table 11:** Ablation results for scallion price forecasting.

Metric	Proposed Model	EMA-PINN-LSTM	LSTM
MAE	0.0581	0.0624	2.2502
MSE	0.0062	0.0068	10.2716
RMSE	0.0792	0.0830	3.2049
R^2^	0.9978	0.9975	−6.2321

**Table 12:** Ablation results for ginger price forecasting.

Metric	Proposed Model	EMA-PINN-LSTM	LSTM
MAE	0.1409	0.1601	1.6960
MSE	0.0350	0.0384	4.2441
RMSE	0.1871	0.1959	2.0601
R^2^	0.9967	0.9964	0.4184

## Data Availability

The daily wholesale price data for garlic, scallion, and ginger were obtained from the National Key Agricultural Products Market Information Platform of the Ministry of Agriculture and Rural Affairs of China. The processed datasets and code supporting the findings of this study are available from the corresponding authors upon reasonable request. Some third-party source data are subject to access restrictions and cannot be publicly redistributed.

## Abbreviations

The following abbreviations are used in this manuscript:

STL	Seasonal-Trend decomposition using LOESS
LOESS	Locally Estimated Scatterplot Smoothing
EMA	Efficient Multi-Scale Attention
PINN	Physics-Informed Neural Network
LSTM	Long Short-Term Memory
PILSTM	Physics-Informed LSTM with Trajectory Regularization
ETO	Exponential-Trigonometric Optimization
SVM	Support Vector Machine
XGBoost	Extreme Gradient Boosting
LightGBM	Light Gradient Boosting Machine
MAE	Mean Absolute Error
MSE	Mean Squared Error
RMSE	Root Mean Squared Error
R^2^	Coefficient of Determination
CPI	Consumer Price Index
M2	Broad Money Supply
USD/CNY	US Dollar to Chinese Yuan Exchange Rate
WTI	West Texas Intermediate
